# Enhancing Performance of the National Field Triage Guidelines Using Machine Learning: Development of a Prehospital Triage Model to Predict Severe Trauma

**DOI:** 10.2196/58740

**Published:** 2024-09-30

**Authors:** Qi Chen, Yuchen Qin, Zhichao Jin, Xinxin Zhao, Jia He, Cheng Wu, Bihan Tang

**Affiliations:** 1 Department of Health Statistics Naval Medical University Shanghai China; 2 School of Medicine Tongji University Shanghai China; 3 Department of Health Management Naval Medical University Shanghai China

**Keywords:** severe trauma, field triage, machine learning, prediction model

## Abstract

**Background:**

Prehospital trauma triage is essential to get the right patient to the right hospital. However, the national field triage guidelines proposed by the American College of Surgeons have proven to be relatively insensitive when identifying severe traumas.

**Objective:**

This study aimed to build a prehospital triage model to predict severe trauma and enhance the performance of the national field triage guidelines.

**Methods:**

This was a multisite prediction study, and the data were extracted from the National Trauma Data Bank between 2017 and 2019. All patients with injury, aged 16 years of age or older, and transported by ambulance from the injury scene to any trauma center were potentially eligible. The data were divided into training, internal, and external validation sets of 672,309; 288,134; and 508,703 patients, respectively. As the national field triage guidelines recommended, age, 7 vital signs, and 8 injury patterns at the prehospital stage were included as candidate variables for model development. Outcomes were severe trauma with an Injured Severity Score ≥16 (primary) and critical resource use within 24 hours of emergency department arrival (secondary). The triage model was developed using an extreme gradient boosting model and Shapley additive explanation analysis. The model’s accuracy regarding discrimination, calibration, and clinical benefit was assessed.

**Results:**

At a fixed specificity of 0.5, the model showed a sensitivity of 0.799 (95% CI 0.797-0.801), an undertriage rate of 0.080 (95% CI 0.079-0.081), and an overtriage rate of 0.743 (95% CI 0.742-0.743) for predicting severe trauma. The model showed a sensitivity of 0.774 (95% CI 0.772-0.776), an undertriage rate of 0.158 (95% CI 0.157-0.159), and an overtriage rate of 0.609 (95% CI 0.608-0.609) when predicting critical resource use, fixed at 0.5 specificity. The triage model’s areas under the curve were 0.755 (95% CI 0.753-0.757) for severe trauma prediction and 0.736 (95% CI 0.734-0.737) for critical resource use prediction. The triage model’s performance was better than those of the Glasgow Coma Score, Prehospital Index, revised trauma score, and the 2011 national field triage guidelines RED criteria. The model’s performance was consistent in the 2 validation sets.

**Conclusions:**

The prehospital triage model is promising for predicting severe trauma and achieving an undertriage rate of <10%. Moreover, machine learning enhances the performance of field triage guidelines.

## Introduction

Trauma is a universal health challenge that places a massive burden on national economies. It causes 4.4 million deaths annually, and an estimated 10% of all years lived with disability [1,2]. The American College of Surgeons Committee on Trauma (ACS-COT) recommends that severe trauma be treated at levels 1 and 2 trauma care facilities [3]. Patients with severe trauma have approximately 25% lower mortality rates when treated at levels 1 or 2 trauma centers than when treated at lower-level or nontrauma centers [4]. Prehospital estimation of injury severity is essential for prehospital triage. It is a critical step for emergency medical service (EMS) providers in making decisions regarding patient destination. Under- and overtriage are incorrect triages. A low-sensitivity triage tool results in many false-negative cases, indicating undertriage and a possible failure in trauma first aid. Conversely, low specificity is associated with a high rate of false-positive cases, indicating overtriage [5].

The national field triage guidelines were initially developed by ACS-COT in 1976 and revised in 2011 and 2021 [6,7]. The national field triage guidelines have been widely implemented in the United States and represent 1 of the few standardized national protocols for EMS. It was developed based on peer-reviewed research, resulting in biased estimates and reduced generalizability [3,8]. A prospective national triage guidelines validation study for identifying high-risk trauma patients found that the guidelines were relatively insensitive in identifying severely injured patients and those requiring early critical resource use [9]. In addition, other triage tools, such as the Glasgow Coma Score (GCS), Prehospital Index (PHI), and revised trauma score (RTS), have not shown ideal predictive performance [5,10-12]. Therefore, it emphasizes the need to improve the prehospital triage tool [13].

Machine learning (ML) development has advanced rapidly in the medical field, notably in trauma medicine, and has demonstrated that the ML model’s predictive ability is significantly better than that of the conventional trauma triage tools for mortality outcomes, hospitalization, and critical care admission [14,15]. Therefore, this study aimed to build a prehospital triage model to predict severe trauma (pTEST) and enhance the performance of the national field triage guideline.

## Methods

### Recruitment

This multisite prediction study was conducted to predict trauma severity during field triage. We developed, validated, and reported our triage model following the TRIPOD (Transparent Reporting of a Multivariable Model for Individual Prognosis or Diagnosis) statement [16], as shown in Multimedia Appendix 1.

### Ethical Considerations

The Naval Medical University Ethics Committee approved the study protocol (reference number NMUEC2022-088). Our study consisted of secondary analyses using the National Trauma Data Bank (NTDB) with primary consent, and the data were anonymized.

### Source of Data and Patients

Data from the NTDB, the largest aggregation of trauma registry data in the United States assembled by the ACS, were used in this study [17]. In 2017, the ACS Trauma Quality Program transitioned to a new technical vendor and redesigned the NTDB infrastructure. The 2017 and 2018 NTDB datasets comprising 2,041,706 patients were used for pTEST model development, hyperparameter tuning, and internal validation. The 2019 NTDB dataset comprising 1,097,190 patients was used for the external validation of the pTEST model.

**Figure 1:**
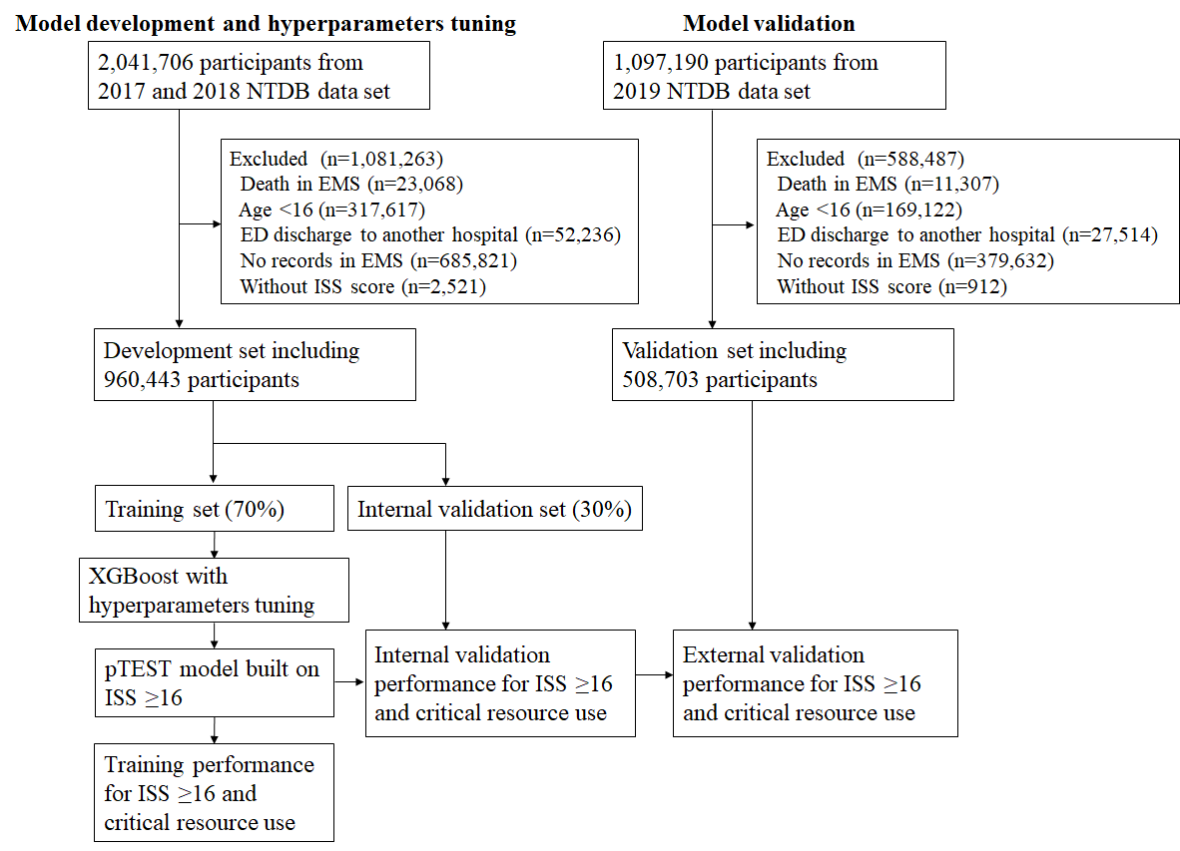
The flowchart of model development and validation. ED: emergency department; EMS: emergency medical service; ISS: Injury Severity Score; NTDB: National Trauma Data Bank; pTEST: prehospital triage model to predict severe trauma.

Notably, all patients with injury, aged 16 years of age or older, and transported by ground or aerial ambulance from the injury scene to any trauma center were potentially eligible. Due to a lack of crucial information on outcomes and predictors, we excluded patients who died in the EMS, patients discharged from the emergency department (ED) to another hospital, and those without any EMS records or an Injury Severity Score (ISS). In total, 960,443 and 508,703 participants were included in the development and validation sets, respectively. Figure 1 shows the patient selection process.

### Outcome and Predictors

Defining “severe trauma” is a challenge in prehospital triage model development. It varies widely across studies. The reference standard (primary outcome) of “severe injury” was considered as an ISS ≥16 as the benchmark to evaluate triage accuracy recommended by the ACS [7,18,19]. The ISS calculated by anatomical criteria is assumed to be consistent with the patient status on the scene and is associated with high mortality [4,19]. However, it does not reflect resource use directly; therefore, we included a composite resource-based early critical resource use measure as the secondary outcome. According to similar studies [9,18], early critical resource use included intubation in the EMS or ED, discharge to the intensive care unit from the ED, surgery for hemorrhage control, interventional radiology procedures, cerebral monitoring, and in-hospital death, all within 24 hours. A detailed definition of severe trauma is provided in Multimedia Appendix 2.

According to a recent field triage protocol review in 2017 [10], the significant predictors of a severely injured patient were age, vital signs, injury patterns, and injury mechanism. In addition, in the US National Guidelines for the Field Triage of Injured Patients in 2011 and 2021 [6,7], age, vital signs, and injury pattern measurements were the field triage’s top priorities. In the 2011 national field triage guidelines, severely injured patients who should be transported preferentially to the highest-level trauma center were identified using the RED criteria, which included 3 vital signs (GCS, systolic blood pressure, and respiratory rate) and 8 injury patterns. During field triage, time is essential, and the number and complexity of hand-collected variables must be limited. Therefore, as the US National Guideline for the Field Triage recommended and recorded in the NTDB, we incorporated 16 candidate variables in EMS for model development, including age at the time of injury (AGEYEARS), GCS eye (EMSGCSEYE), GCS motor (EMSGCSMOTOR), GCS verbal (EMSGCSVERBAL), systolic blood pressure (EMSSBP), oxygen saturation (EMSPULSEOXIMETRY), respiratory rate (EMSRESPIRATORYRATE), pulse rate (EMSPULSERATE), penetrating injuries (TCCPEN), chest wall instability (TCCCHEST), long-bone fractures (TCCLONGBONE), crushed extremity (TCCCRUSHED), amputation (TCCAMPUTATION), pelvic fracture (TCCPELVIC), skull fracture (TCCSKULLFRACTURE), and paralysis (TCCPARALYSIS). The detailed definitions of candidate variables are listed in Multimedia Appendix 3.

### Model Development

The pTEST model was developed using the extreme gradient boosting model (XGBoost) and Shapley additive explanation analysis (SHAP). XGBoost is a novel boosting tree-based ensemble algorithm through which new models are created to predict residuals or errors of prior models and then combined to make a final prediction [20]. Recently, XGBoost has been widely used in ML due to its outstanding prediction performance, ability to use continuous and categorical inputs, lack of data preprocessing, imbalanced data handling capacity, high internal optimization, and relatively modest computational costs [21].

Patients in the development set from the 2017 and 2018 NTDB datasets were randomly grouped into training (70%) and internal validation (30%) sets for model development. Using a grid search, a 10-fold cross-validation process was used on the training set for hyperparameter tuning. The goal of hyperparameter tuning is to find the values that lead to the best-predicted performance. The optimal values of hyperparameters were learning rate=0.04071151, minimum loss reduction required to make a further partition=20.36485, maximum tree depth=14, minimum sum of instance weights needed in a leaf node=39, maximum number of boosting iterations=1051, and subsample ratio of the training instance=0.7763707, with the other hyperparameters set to default. In addition to the training set, the model’s reproducibility, transportability, and generalizability were evaluated using internal and external validation sets.

Missing values are an essential concern in trauma triage because there may not always be time to measure critical variables. The absent proportions of the training, internal validation, and external validation sets are shown in Multimedia Appendix 4, with all variables, except pulse oximetry, missing below 6%. XGBoost supports branch directions for predictors with missing values, creating an advantage in real-world situations where XGBoost can still achieve individual prediction without complete prehospital data.

To gain insight into the risk prediction model, we investigated different predictors’ contributions based on Shapley values using SHAP, a game theory concept introduced in the 1950s [22,23]. A predictor’s SHAP value can be positive or negative, suggesting an increased or decreased probability of severe trauma, respectively. In our study, the SHAP values were visualized at global (dataset level) and local (patient-specific) levels to investigate the predictors’ impact. XGBoost and SHAP were implemented using the R (R Core Team) packages *tidy models* and *shapviz*.

### Statistical Analysis Methods

For the sample size calculation, the prevalence of events was set at 17.8%, and the number of predictors was 16 based on the development set. The area under the curve (AUC) of the optimal prehospital triage model in a previous study was 0.68 [11], and our pTEST model was expected to achieve an AUC of 0.7. At least 1871 patients were required for model development or validation using the R package *pmsampsize* [24].

Continuous data are presented as mean and SD, and categorical data are presented as frequencies and percentages (%). The 2-tailed *t* test was used to evaluate the differences in continuous data, which followed a normal distribution and variance homogeneity; otherwise, the Wilcoxon rank-sum test was used. The differences in categorical data were evaluated using the Pearson chi-square test. The area under the receiver operating characteristic curve was calculated to assess model discrimination. The AUCs between models were compared using the DeLong test. The best thresholds of the models were determined by maximizing the Youden index, and performance metrics, including sensitivity, specificity, accuracy, positive predictive value (PPV), and negative predictive value (NPV), were calculated. Performance metrics 95% CIs were calculated using 500 bootstrap replicates. In addition, the pTEST model is intended to identify severe trauma that requires high sensitivity and NPV to rule it out. Sensitivity and specificity are inversely proportional and a tradeoff needs to be made between sensitivity and specificity. Therefore, the sensitivities and NPV of the different models were compared using a 0.5 specificity as in the previous studies [25,26].

Our study defined the over- and undertriage rates. Overtriage rate (1-PPV) is equal to the number of patients with negative outcomes (ISS <16 or no critical resource use) predicted as positive outcomes divided by the total number of predicted positive outcomes. The undertriage rate (1-NPV) is equal to the number of patients with positive outcomes predicted as negative outcomes divided by the total number of predicted negative outcomes.

In addition, a calibration plot and Brier score were generated to assess how closely the predicted probability approximated the actual probability. The clinical benefit of the models was evaluated using a decision curve analysis method. The discrimination, calibration, and clinical benefit of the pTEST were compared with the GCS, PHI, RTS, and RED criteria of the 2011 National Field Triage Guidelines. Statistical significance was set at *P*<.05. All statistical analyses were performed using the R software (version 4.3.1).

## Results

### Patient Characteristics

Table 1 shows the patients’ baseline characteristics in the training, internal validation, and external validation sets. In these 3 sets, severe trauma proportions were 17.80% (119,690/672,309), 17.80% (51,296/288,134), and 17.08% (86,902/508,703), respectively, and critical resource use proportions were 29.36% (177,570/604,806), 29.56% (76,604/259,148), and 28.17% (129,551/459,843), respectively. Notably, most variables showed statistically significant differences among the 3 sets for large sample sizes, but the differences were minimal. The demographic characteristics, vital signs, and injury patterns of nonsevere and severe trauma are listed in Multimedia Appendices 5-7, and the attributes of noncritical and critical resource users are listed in Multimedia Appendices 8-10. Severe trauma and critical resource users are usually male, air-transported, taken to higher-level trauma centers, and have extreme trauma patterns.

**Table 1:** Baseline characteristics of the patients from the training set, internal validation set, and external validation set.

Characteristics		Training set (n=672,309)	Internal validation set (n=288,134)	External validation set (n=508,703)	*P* value	
**Sex (male), n (%)**		403,434 (60.01)	172,821 (59.98)	300,711 (59.12)	<.001	
**Transport mode, n (%)**						<.001
	Ground	622,489 (92.59)	266,622 (92.53)	474,645 (93.30)		
	Helicopter	48,464 (7.21)	20,911 (7.26)	33,346 (6.56)		
	Fixed-wing	1356 (0.20)	601 (0.21)	712 (0.14)		
**Trauma center level, n (%)**						<.001
	Level 1	275,723 (55.75)	118,199 (55.72)	206,563 (54.39)		
	Level 2	179,419 (36.28)	77,005 (36.30)	142,433 (37.50)		
	Level 3	39,397 (7.97)	16,944 (7.99)	30,819 (8.11)		
**TCCPEN^a^ (yes), n (%)**		26,987 (4.01)	11,319 (3.93)	17,658 (3.47)	<.001	
**TCCCHEST^b^ (yes), n (%)**		4295 (0.64)	1825 (0.63)	3216 (0.63)	.89	
**TCCLONGBONE^c^ (yes), n (%)**		4598 (0.68)	1867 (0.65)	3213 (0.63)	.002	
**TCCCRUSHED^d^ (yes), n (%)**		3188 (0.47)	1374 (0.48)	2712 (0.53)	<.001	
**TCCAMPUTATION^e^ (yes), n (%)**		832 (0.12)	372 (0.13)	613 (0.12)	.58	
**TCCPELVIC^f^ (yes), n (%)**		7495 (1.11)	3346 (1.16)	5852 (1.15)	.07	
**TCCSKULLFRACTURE^g^ (yes), n (%)**		5127 (0.76)	2237 (0.78)	4087 (0.80)	.04	
**TCCPARALYSIS^h^ (yes), n (%)**		4379 (0.65)	1887 (0.65)	3077 (0.60)	.003	
**ISS^i^ score (≥16), n (%)**		119,690 (17.80)	51,296 (17.80)	86,902 (17.08)	<.001	
**Surgery for hemorrhage control (yes), n (%)**		14,714 (2.45)	6441 (2.50)	10,886 (2.37)	.002	
**Cerebral monitor (yes), n (%)**		8527 (1.42)	3563 (1.38)	5819 (1.27)	<.001	
**Interventional radiology procedures (yes), n (%)**		5154 (0.86)	2182 (0.85)	3848 (0.84)	.59	
**Discharge to the ICU^j^ from ED^k^ (yes), n (%)**		142,422 (21.46)	61,384 (21.58)	105,670 (21.06)	<.001	
**In-hospital death within 24 hours (yes), n (%)**		9303 (1.38)	3987 (1.38)	6371 (1.25)	<.001	
**Intubation in the EMS^l^ or ED (yes), n (%)**		72,755 (10.82)	31,080 (10.79)	49,779 (9.79)	<.001	
**Critical resource use (yes), n (%)**		177,570 (29.36)	76,604 (29.56)	129,551 (28.17)	<.001	
**RED criteria (yes), n (%)**		87,577 (13.03)	37,448 (13.00)	62,194 (12.23)	<.001	
**Age (years), mean (SD)**		53.12 (21.86)	53.22 (21.84)	54.40 (21.77)	<.001	
**EMSSBP^m^ (mm Hg), mean (SD)**		139.89 (28.37)	139.92 (28.42)	140.74 (28.62)	<.001	
**EMSPULSERATE^n^ (n/minute), mean (SD)**		90.58 (20.31)	90.58 (20.29)	90.34 (20.41)	<.001	
**EMSRESPIRATORYRATE^o^ (n/minute), mean (SD)**		18.42 (4.73)	18.44 (4.79)	18.46 (4.82)	.08	
**EMSPULSEOXIMETRY^p^, mean (SD)**		96.26 (5.50)	96.26 (5.48)	96.19 (5.44)	<.001	
**EMSGCSEYE^q^, mean (SD)**		3.81 (0.65)	3.81 (0.65)	3.82 (0.63)	<.001	
**EMSGCSVERBAL^r^, mean (SD)**		4.60 (0.95)	4.60 (0.95)	4.61 (0.93)	.02	
**EMSGCSMOTOR^s^, mean (SD)**		5.73 (0.97)	5.73 (0.97)	5.75 (0.94)	<.001	
**EMSTOTALGCS^t^, mean (SD)**		14.12 (2.44)	14.12 (2.43)	14.17 (2.36)	<.001	
**Total time spent in ED (minutes), mean (SD)**		189.94 (150.35)	207.75 (400.72)	207.68 (1355.03)	<.001	
**Length of stay (days), mean (SD)**		6.20 (9.67)	6.21 (8.51)	6.21 (8.38)	.001	
**ISS score, mean (SD)**		9.69 (8.37)	9.69 (8.32)	9.55 (8.20)	<.001	
**PHI^u^ score, mean (SD)**		1.32 (2.23)	1.32 (2.23)	1.31 (2.22)	.26	
**RTS^v^ score, mean (SD)**		11.73 (0.88)	11.73 (0.89)	11.74 (0.86)	.045	

^a^TCCPEN: penetrating injuries.

^b^TCCCHEST: chest wall instability.

^c^TCCLONGBONE: long-bone fractures.

^d^TCCCRUSHED: crushed extremity.

^e^TCCAMPUTATION: amputation.

^f^TCCPELVIC: pelvic fracture.

^g^TCCSKULLFRACTURE: skull fracture.

^h^TCCPARALYSIS: paralysis.

^i^ISS: Injury Severity Score.

^j^ICU: intensive care unit.

^k^ED: emergency department.

^l^EMS: emergency medical service.

^m^EMSSBP: systolic blood pressure.

^n^EMSPULSERATE: pulse rate.

^o^EMSRESPIRATORYRATE: respiratory rate.

^p^EMSPULSEOXIMETRY: oxygen saturation.

^q^EMSGCSEYE: Glasgow Coma Score eye.

^r^EMSGCSVERBAL: Glasgow Coma Score verbal.

^s^EMSGCSMOTOR: Glasgow Coma Score motor.

^t^EMSTOTALGCS: Glasgow Coma Score total.

^u^PHI: Prehospital Index.

^v^RTS: revised trauma score.

### Model Performance

For predicting severe trauma, we compared the performance metrics of the other models at the same specificity fixed at a moderate number of 0.5. The pTEST model showed a higher sensitivity of 0.799 (95% CI 0.797-0.801), a lower undertriage rate of 0.080 (95% CI 0.079-0.081), and a lower overtriage rate of 0.743 (95% CI 0.742-0.743) in the training set (Table 2). In addition, for critical resource use prediction fixed at a specificity of 0.5, the pTEST model showed a higher sensitivity of 0.774 (95% CI 0.772-0.776), lower undertriage rate of 0.158 (95% CI 0.157-0.159), and lower overtriage rate of 0.609 (95% CI 0.608-0.609) than the other models in the training set (Multimedia Appendix 11). We validated the pTEST model performance using 2 validation sets and obtained consistent results (Table 2 and Multimedia Appendix 11). The model performance metrics for predicting severe trauma and critical resource use at the best thresholds with the maximum Youden index are listed in Multimedia Appendices 12 and 13, demonstrating a higher pTEST Youden index than other models.

**Table 2:** Model performance metrics for predicting severe trauma fixed at a specificity of 0.5.

Prediction tool			Specificity		Sensitivity (95% CI)	Accuracy, AUC (95% CI)		Undertriage rate (1-NPV), AUC (95% CI)		Overtriage rate (1-PPV), AUC (95% CI)		Youden index
**Training set**												
	pTEST^a^	0.500		0.799 (0.797-0.801)		0.553 (0.553-0.554)	0.080 (0.079-0.081)		0.743 (0.742-0.743)		1.299	
	GCS^b^	0.500		0.682 (0.680-0.684)		0.532 (0.532-0.532)	0.119 (0.119-0.120)		0.774 (0.774-0.775)		1.182	
	PHI^c^	0.500		0.711 (0.709-0.713)		0.536 (0.536-0.537)	0.107 (0.107-0.108)		0.772 (0.771-0.772)		1.211	
	RTS^d^	0.500		0.634 (0.632-0.636)		0.523 (0.523-0.523)	0.132 (0.132-0.133)		0.791 (0.791-0.792)		1.134	
	RED criteria	0.500		0.620 (0.619-0.621)		0.521 (0.521-0.522)	0.141 (0.141-0.142)		0.788 (0.788-0.789)		1.120	
**Internal validation set**												
	pTEST	0.500		0.794 (0.791-0.798)		0.552 (0.552-0.553)	0.082 (0.081-0.083)		0.744 (0.743-0.745)		1.294	
	GCS	0.500		0.682 (0.680-0.685)		0.532 (0.532-0.533)	0.120 (0.119-0.120)		0.774 (0.773-0.775)		1.182	
	PHI	0.500		0.710 (0.707-0.714)		0.536 (0.536-0.537)	0.108 (0.106-0.109)		0.772 (0.771-0.773)		1.210	
	RTS	0.500		0.633 (0.631-0.636)		0.523 (0.523-0.523)	0.132 (0.132-0.133)		0.791 (0.791-0.792)		1.133	
	RED criteria	0.500		0.620 (0.619-0.622)		0.521 (0.521-0.522)	0.141 (0.141-0.142)		0.788 (0.788-0.789)		1.120	
**External validation set**												
	pTEST	0.500		0.794 (0.792-0.797)		0.550 (0.550-0.551)	0.078 (0.077-0.079)		0.753 (0.753-0.754)		1.294	
	GCS	0.500		0.681 (0.679-0.683)		0.531 (0.530-0.531)	0.115 (0.115-0.116)		0.783 (0.782-0.783)		1.181	
	PHI	0.500		0.709 (0.706-0.711)		0.535 (0.534-0.535)	0.103 (0.103-0.104)		0.781 (0.780-0.782)		1.209	
	RTS	0.500		0.633 (0.631-0.635)		0.522 (0.522-0.522)	0.127 (0.126-0.128)		0.799 (0.799-0.800)		1.133	
	RED criteria	0.500		0.616 (0.615-0.618)		0.520 (0.520-0.520)	0.137 (0.136-0.137)		0.798 (0.797-0.798)		1.116	

^a^pTEST: prehospital triage model to predict severe trauma.

^b^GCS: Glasgow Coma Score.

^c^PHI: Prehospital Index.

^d^RTS: revised trauma score.

**Figure 2:**
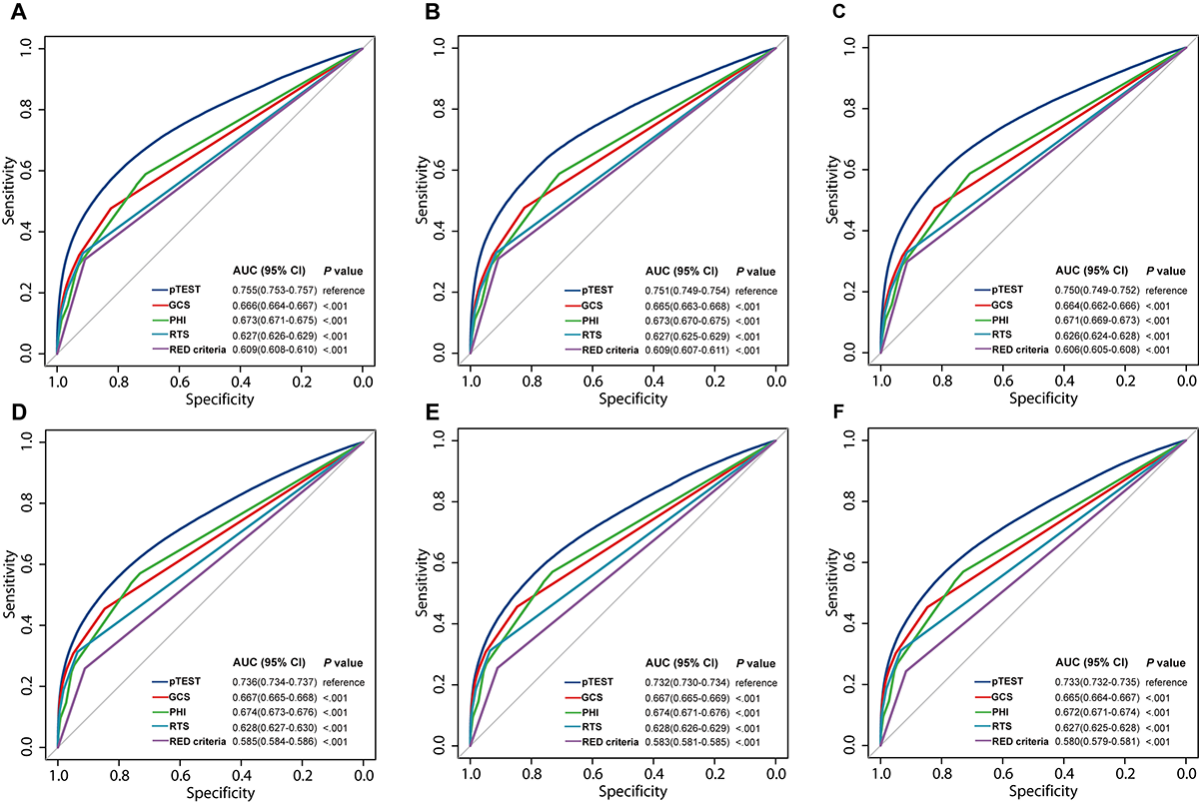
The ROC curves of 5 models. (A) Predicting severe trauma in the training set. (B) Predicting severe trauma in the internal validation set. (C) Predicting severe trauma in the external validation set. (D) Predicting critical resource use in training set. (E) Predicting critical resource use in the internal validation set. (F) Predicting critical resource use in the external validation set. AUC: area under the curve; GCS: Glasgow Coma Score; PHI: Prehospital Index; pTEST: prehospital triage model to predict severe trauma; ROC: receiver operating characteristic; RTS: revised trauma score.

In Figure 2, pTEST AUCs for severe trauma prediction were 0.755 (95% CI 0.753-0.757), 0.751 (95% CI 0.749-0.754), and 0.750 (95% CI 0.749-0.752) in training, internal validation, and external validation sets, respectively, and the AUCs for predicting critical resource use were 0.736 (95% CI 0.734-0.737), 0.732 (95% CI 0.730-0.734), and 0.733 (95% CI 0.732-0.735), respectively, demonstrating better discrimination ability than GCS, PHI, RTS, and RED criteria. Multimedia Appendix 14 depicts the pTEST model predicted outcome probability as a waterfall plot. The calibration curves in Multimedia Appendix 15 show that the severe trauma predicted probability and pTEST critical resource use agreed with the proportion observed using the smallest Brier score. In Multimedia Appendix 16, pTEST provides a consistently higher net benefit across a broad range of risk thresholds (10%-100%) than the 2 default strategies and other models.

### Model Interpretation

**Figure 3:**
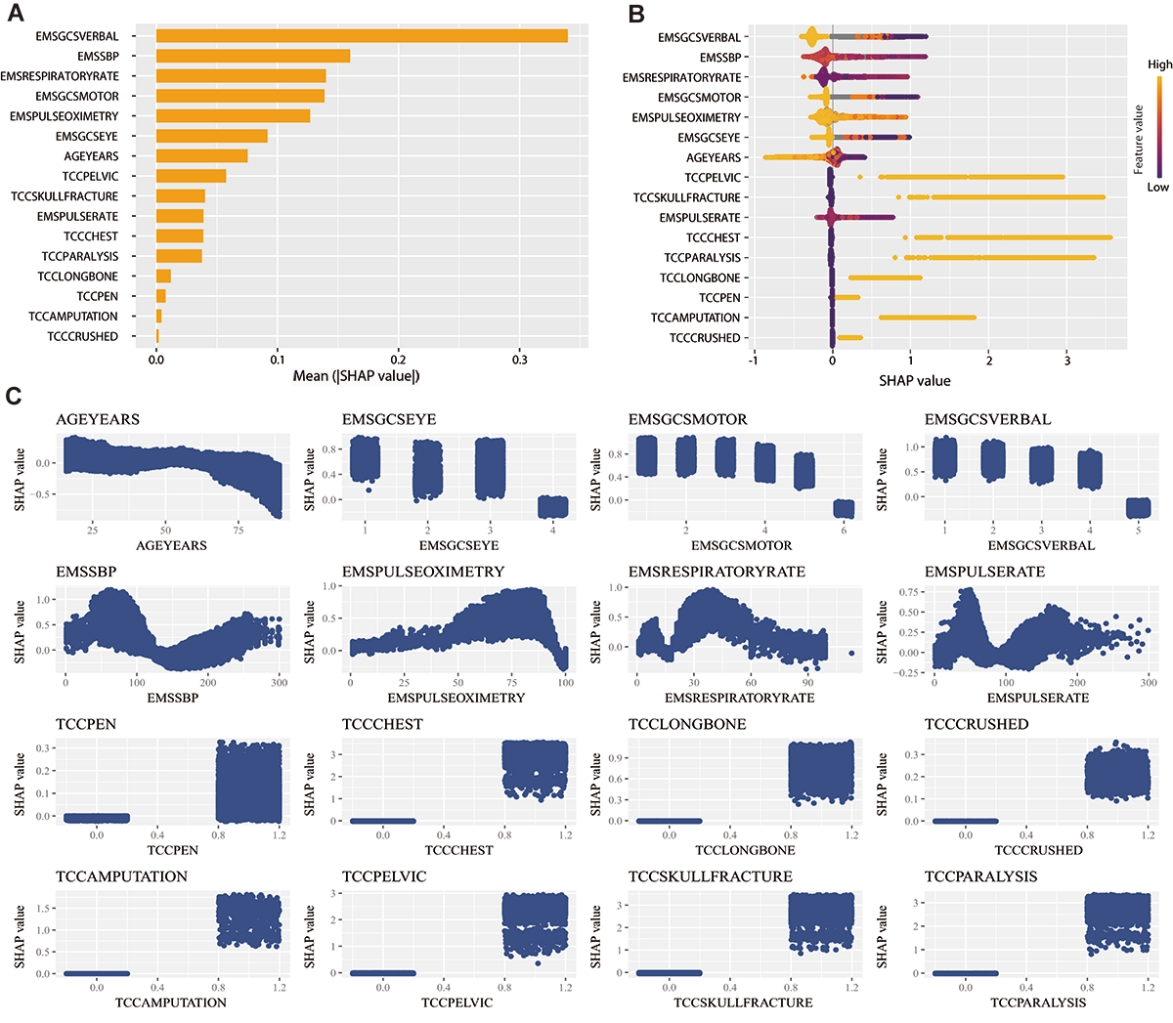
The global model explanation for predicting severe trauma by the SHAP method in the training set. (A) SHAP summary bar plot of the average SHAP value for each variable. (B) SHAP summary dot plot. In each variable, a dot is made for each single patient, representing the SHAP value of this variable. The colors of the dots demonstrate the actual values of the features, and the dots are stacked vertically to show density. (C) SHAP dependence plot. Each dependence plot shows the association between the actual value and the SHAP value of the variable, and each dot represents a single patient. AGEYEARS: age at the time of injury; EMSGCSEYE: Glasgow Coma Score eye; EMSGCSMOTOR: Glasgow Coma Score motor; EMSGCSVERBAL: Glasgow Coma Score verbal; EMSSBP: systolic blood pressure; EMSPULSEOXIMETRY: oxygen saturation; EMSRESPIRATORYRATE: respiratory rate; EMSPULSERATE: pulse rate; SHAP: Shapley additive explanation analysis; TCCPEN: penetrating injuries; TCCCHEST: chest wall instability; TCCLONGBONE: long-bone fractures; TCCCRUSHED: crushed extremity; TCCAMPUTATION: amputation; TCCPELVIC: pelvic fracture; TCCSKULLFRACTURE: skull fracture; TCCPARALYSIS: paralysis.

As shown in the SHAP summary plots (Figure 3A), the contributions of the variables to the pTEST model for severe trauma prediction were evaluated using the average absolute SHAP values; the top 5 important variables were EMSGCSVERBAL, EMSSBP, EMSRESPIRATORYRATE, EMSGCSMOTOR, and EMSPULSEOXIMETRY. Figure 3B lists the impact of the different variables illustrated by the SHAP values for severe trauma prediction. Figure 3C shows each variable’s SHAP values versus measured values. Figure 3B, C shows that the higher the EMSGCSVERBAL score, the lower the probability of severe trauma (“negative” impact). Similarly, AGEYEARS, EMSGCSEYE, EMSGCSMOTOR, and EMSPULSEOXIMETRY negatively contributed to the predicted probability. In contrast, 8 injury patterns contributed positively to the predicted probability. The SHAP summary and dependence plots of the pTEST model for critical resource use prediction are shown in Multimedia Appendix 17. The personalized feature attributes for 2 representative patients with and without severe trauma in the training set are provided in Multimedia Appendix 18.

### Subgroup Analysis

In Multimedia Appendix 19, subgroup analyses were performed according to age, sex, transport mode, trauma type, and prehospital time. The pTEST model AUCs for severe trauma prediction in patients 60 years of age or older were relatively low at 0.717 (95% CI 0.714-0.720), 0.712 (95% CI 0.708-0.717), and 0.710 (95% CI 0.707-0.714) in the 3 sets, respectively. The AUCs in patients with penetrating injuries were relatively high at 0.815 (95% CI 0.810-0.820), 0.810 (95% CI 0.802-0.817), and 0.810 (95% CI 0.805-0.816) in the 3 sets, respectively. The high proportion of severe trauma in helicopter-transported patients (approximately 41%) led to a high undertriage rate (>0.2) and a low overtriage rate (<0.5). Multimedia Appendix 20 illustrates the pTEST model performance in critical resource use prediction in different subgroups.

## Discussion

### Principal Findings

In this multisite, large-sample study, we present a prehospital trauma triage tool, pTEST, for severe trauma prediction in EMS. To our knowledge, this is the first study combining ML with national triage guidelines. The pTEST performed optimally in internal and external validations. In addition, its diagnostic accuracy was evaluated using anatomical and resource-based outcomes, and the resource-based outcome is a better alternative to determine the need for specialized trauma care [27]. Furthermore, the pTEST was developed based on national triage guidelines, a globally adopted standard in many organizations. Therefore, the pTEST model can be conveniently applied in EMS practice and may have global relevance.

### Comparison With Previous Studies

Consistent with previous studies [8], we also demonstrated the poor performance of the RED criteria from the national triage guidelines. Globally, all triage guidelines are based on a criteria checklist, including vital signs, injury type, and mechanism of injury [7,28,29]. These guidelines are simplistic—patients meeting any 1 of the criteria should be transported to the highest-level trauma center. In reality, there was an interaction among variables and nonlinear effects of continuous variables (Figure 3C). XGBoost, as a nonlinear ensemble method, can train a more accurate classifier from several weak classifiers and has other benefits, such as dealing with missing values and interaction, avoiding overfitting, and accelerating the training speed by parallel calculation [30]. Our study incorporated XGBoost into national field triage guidelines and developed the pTEST model to enhance performance. The pTEST model included age, 7 vital signs, and 8 injury patterns. We did not perform further variable selection because these variables are the most important in the national field triage guidelines, and the overall number is moderate for field triage. The pTEST model did not achieve an undertriage rate of <5% or an overtriage rate of <35%, as the ACS Committee on Trauma targeted. However, 2 aspects must be noted. First, the definition of the undertriage rate by the ACS is different from that used in our study. For example, the undertriage rate in ACS is equal to the number of patients with ISS ≥16 transported to a low-level trauma center or nontrauma center divided by the total number of patients transported to a low-level trauma center or nontrauma center [19]. In contrast, the undertriage rate in our study is equal to the number of patients with ISS ≥16 predicted as ISS <16 divided by the total number of patients predicted ISS <16. In the national field triage guideline, the patients predicted as ISS <16 can be transported to a low-level or nontrauma center. If the patients with predicted ISS <16 and ≥16 are transferred to low and high-level trauma centers, respectively, then the undertriage rate in our study is the same as that in ACS, but this is unlikely in practice. Second, the under- and overtriage rates are affected by severe trauma proportion. Since most (>90%) of our study population came from level 1 and 2 trauma centers, the severe trauma proportion was approximately 18%, resulting in undertriage rate overestimation and overtriage rate underestimation. Based on previous studies with good sample representativeness [9], we assumed that the proportion of severe trauma was 3%. Keeping the sensitivity (0.794) and specificity (0.5) of the pTEST model and the sample size (n=508,703) in the external validation set unchanged, the under- and overtriage rates were 1.26% and 95.3%, respectively, in the external validation set (Multimedia Appendix 20), meeting the ACS undertriage rate target.

The pTEST model and the national field triage guidelines in subgroup analyses were particularly insensitive among older adults [9]. Possible explanations include different physiological responses to injury [31], medication use that potentially worsens injury [32], high prevalence of frailty, and comorbidities [33]. Previous studies have explored elderly-specific triage criteria [15,34,35]. The pTEST model performed better for penetrating traumas. Notably, several studies have found that penetration is a strong severe trauma predictor, and severely penetrated injured patients are more easily recognized [10]. The undertriage rate was high in patients transported using helicopters. Patients with a high proportion of severe trauma, such as those experiencing large-scale casualties, should be transferred to high-level trauma centers without field triage to reduce the undertriage rate.

Previous studies have reported controversial results regarding the use of ML in medical prediction issues [36]. Overall, in studies with a limited number of predictors, ML does not demonstrate advantages over traditional models, such as logistic regression [37], whereas, for studies with many predictors, advanced ML may have an advantage [38]. A recent review, including 14 studies, demonstrated that the predictive ability of ML-based models was significantly better than that of conventional trauma triage tools for outcomes of mortality, hospitalization, and critical care admission, and XGBoost was the most commonly used ML algorithm [14]. In this study, the relatively large number of predictors and sufficient amount of data tended to favor ML applications. We built the pTEST model using XGBoost but did not evaluate other ML methods. We believe an excellent model can be created using a large sample size, an advanced ML method, and robust hyperparameter tuning. In addition, we minimized the risk of chance findings and overfitting by avoiding exploring other modeling strategies.

### Limitations

This study had some limitations. First, most of our study population were from level 1 and 2 trauma centers, and the proportion of patients with severe trauma (approximately 18%) was significantly higher than that of all prehospital trauma patients (approximately 3%) [9]. However, unlike PPV and NPV, the sensitivity, specificity, and AUC of the pTEST model were not affected by the proportion of severe trauma, and the high sensitivity, specificity, and AUC objectively reflect the pTEST model’s good performance. In addition, some emergency resources may be unavailable in low-level trauma and nontrauma centers [39], and samples from high-level trauma centers make it possible to evaluate the pTEST model with critical resource use as the end point. Second, the pTEST model was not developed into a software application, as in other studies [40], because software development requires adaptation to existing information systems in EMS, which is a complex project. However, in the future, EMS providers can develop software based on available data and programs. Third, the 2017-2019 NTDB data followed the 2011 national field triage guidelines, and the latest guidelines have been revised in 2021. An additional “active bleeding” has been added to high-risk trauma types [7]. The new guidelines will take several years to be implemented, and our model must be further validated and updated as necessary.

### Conclusions

We constructed a prehospital triage model, pTEST, to predict severe trauma and achieved an undertriage rate of <10%. Moreover, our study demonstrated that ML is a promising method for enhancing field triage guidelines performance. In the future, we will validate our pTEST model using populations from different countries and casualty backgrounds. In addition, software must be developed to increase user convenience of the pTEST model in the EMS.

## Appendix

Multimedia Appendix 1 TRIPOD (Transparent Reporting of a Multivariable Prediction Model for Individual Prognosis or Diagnosis) checklist.

Multimedia Appendix 2 Detailed definition of severe trauma and critical resource use.

Multimedia Appendix 3 The candidate variables for model development.

Multimedia Appendix 4 The missing proportion of the candidate variables for model development. (A) The missing proportion in training set. (B) The missing proportion in internal validation set. (C) The missing proportion in external validation set.

Multimedia Appendix 5 The demographic characteristics, vital signs, and injury patterns between nonsevere traumas and severe traumas in training set.

Multimedia Appendix 6 The demographic characteristics, vital signs, and injury patterns between nonsevere traumas and severe traumas in internal validation set.

Multimedia Appendix 7 The demographic characteristics, vital signs, and injury patterns between nonsevere traumas and severe traumas in external validation set.

Multimedia Appendix 8 The demographic characteristics, vital signs, and injury patterns between no critical resource users and critical resource users in training set.

Multimedia Appendix 9 The demographic characteristics, vital signs, and injury patterns between no critical resource users and critical resource users in internal validation set.

Multimedia Appendix 10 The demographic characteristics, vital signs, and injury patterns between no critical resource users and critical resource users in external validation set.

Multimedia Appendix 11 Model performance metrics for predicting critical resource use fixed at a specificity of 0.5.

Multimedia Appendix 12 Model performance metrics for predicting severe trauma at the best thresholds with maximum Youden index.

Multimedia Appendix 13 Model performance metrics for predicting critical resource use at the best thresholds with maximum Youden index.

Multimedia Appendix 14 Waterfall plot for the predicted probability of pTEST model related to the outcome. (A) Predicted probability of severe trauma in training set. (B) Predicted probability of severe trauma in internal validation set. (C) Predicted probability of severe trauma in external validation set. (D) Predicted probability of critical resource use in training set. (E) Predicted probability of critical resource use in internal validation set. (F) Predicted probability of critical resource use in external validation set. pTEST: prehospital triage model to predict severe trauma.

Multimedia Appendix 15 Calibration curves of 5 models. (A) Predicting severe trauma in training set. (B) Predicting severe trauma in internal validation set. (C) Predicting severe trauma in external validation set. (D) Predicting critical resource use in training set. (E) Predicting critical resource use in internal validation set. (F) Predicting critical resource use in external validation set.

Multimedia Appendix 16 DCA curves of 5 models. (A) Predicting severe trauma in training set. (B) Predicting severe trauma in internal validation set. (C) Predicting severe trauma in external validation set. (D) Predicting critical resource use in training set. (E) Predicting critical resource use in internal validation set. (F) Predicting critical resource use in external validation set. DCA: decision curve analysis.

Multimedia Appendix 17 Global model explanation for predicting critical resource use by the SHAP method in training set. (A) SHAP summary bar plot of the average SHAP value for each variable. (B) SHAP summary dot plot. (C) SHAP dependence plot. SHAP: Shapley additive explanation analysis.

Multimedia Appendix 18 Local model explanation for predicting severe trauma by the SHAP method in training set. (A) Waterfall plot of risks contributed by each variable for a patient at high risk of severe trauma. (B) Waterfall plot for a patient at low risk of severe trauma. SHAP: Shapley additive explanation analysis.

Multimedia Appendix 19 The performance of the pTEST model for predicting severe trauma in different subgroups. pTEST: prehospital triage model to predict severe trauma.

Multimedia Appendix 20 The performance of the pTEST model for predicting critical resource use in different subgroups. The undertriage and overtriage rates of the pTEST in external validation set with the fixed sensitivity, specificity, and sample size. pTEST: prehospital triage model to predict severe trauma.

## Abbreviations

ACS-COT: American College of Surgeons Committee on Trauma
AUC: area under the curve
ED: emergency department
EMS: emergency medical service
GCS: Glasgow Coma Score
ISS: Injury Severity Score
ML: machine learning
NPV: negative predictive value
NTDB: National Trauma Data Bank
PHI: Prehospital Index
PPV: positive predictive value
pTEST: prehospital triage model to predict severe trauma
RTS: revised trauma score
SHAP: Shapley additive explanation analysis
TRIPOD: Transparent Reporting of a Multivariable Prediction Model for Individual Prognosis or Diagnosis
XGBoost: extreme gradient boosting model

## Data Availability

The datasets generated and analyzed during this study are available in ACS Trauma Quality Programs.
