# Supplementary material for: Enhancing Performance of the National Field Triage Guidelines Using Machine Learning: Development of a Prehospital Triage Model to Predict Severe Trauma
Source: J Med Internet Res. 2024 Sep 30;26:e58740. doi: 10.2196/58740 (PMC11474124; doi:10.2196/58740)
Supplement: Multimedia Appendix 2 [file jmir_v26i1e58740_app2.docx]

| **Outcome** | **Defination** | **Variable in NTDB** |
| --- | --- | --- |
| (1) Severe trauma | AIS derived ISS ≥16 | ISS_05 |
| (2) Critical resource use |  |  |
| Intubation in the EMS or ED | Intubation in the EMS or ED | GCSQ_INTUBATED  ICDPROCEDURECODE (ICD–10–PCS: 0BH17EZ，0BH18EZ) |
| Discharge to the intensive care unit from ED | ICU admission after discharge from the emergency department | EDDISCHARGEDISPOSITION |
| Surgery for Hemorrhage Control within 24 h | Surgery for hemorrhage control within 24 h in the patients with transfused packed red blood cells, including laparotomy, thoracotomy, sternotomy, extremity surgery, neck surgery, traumatic amputation, skin/soft tissue surgery, extraperitoneal pelvic packing | HMRRHGCTRLSURGTYPE |
| Interventional radiology procedures within 24 h | Interventional angiogram with or without embolization/ stenting within 24 h in the patients with transfused packed red blood cells | ANGIOGRAPHY |
| cerebral monitor within 24 h | All placed cerebral monitors, including ventriculostomy, subarachnoid bolt, camino bolt, external ventricular drain, licox monitor, jugular venous bulb | ICPEVDRAIN  ICPPARENCH  ICPO2MONITOR  ICPJVBULB |
| In-hospital death within 24 h | Death in ED or hospital within 24 | HOSPDISCHARGEDISPOSITION  EDDISCHARGEDISPOSITION |
