# Supplementary material for: Enhancing Performance of the National Field Triage Guidelines Using Machine Learning: Development of a Prehospital Triage Model to Predict Severe Trauma
Source: J Med Internet Res. 2024 Sep 30;26:e58740. doi: 10.2196/58740 (PMC11474124; doi:10.2196/58740)
Supplement: Multimedia Appendix 3 [file jmir_v26i1e58740_app3.docx]

| **Candidate variables** | **Variable in NTDB*** |
| --- | --- |
| The patient's age at the time of injury (best approximation) | AGEYEARS |
| First recorded Glasgow Coma Score (Eye) measured at the scene of injury | EMSGCSEYE |
| First recorded Glasgow Coma Score (Motor) measured at the scene of injury | EMSGCSMOTOR |
| First recorded Glasgow Coma Score (Verbal) measured at the scene of injury | EMSGCSVERBAL |
| First recorded systolic blood pressure measured at the scene of injury | EMSSBP |
| First recorded oxygen saturation measured at the scene of injury (expressed as a percentage) | EMSPULSEOXIMETRY |
| First recorded respiratory rate measured at the scene of injury (expressed as a number per minute) | EMSRESPIRATORYRATE |
| First recorded pulse measured at the scene of injury (palpated or auscultated), expressed as a number  per minute | EMSPULSERATE |
| Trauma Center Critera: All penetrating injuries to head, neck, torso, and extremities proximal to elbow or knee | TCCPEN |
| Trauma Center Critera: Chest wall instability or deformity (e.g., flail chest) | TCCCHEST |
| Trauma Center Critera: Two or more proximal long-bone fractures | TCCLONGBONE |
| Trauma Center Critera: Crushed, degloved, mangled, or pulseless extremity | TCCCRUSHED |
| Trauma Center Critera: Amputation proximal to wrist or ankle | TCCAMPUTATION |
| Trauma Center Critera: Pelvic fracture | TCCPELVIC |
| Trauma Center Critera: Open or depressed skull fracture | TCCSKULLFRACTURE |
| Trauma Center Critera: Paralysis | TCCPARALYSIS |

* As an abbreviation in the article
