# Supplementary figures and images for: Enhancing Performance of the National Field Triage Guidelines Using Machine Learning: Development of a Prehospital Triage Model to Predict Severe Trauma
Source: J Med Internet Res. 2024 Sep 30;26:e58740. doi: 10.2196/58740 (PMC11474124; doi:10.2196/58740)

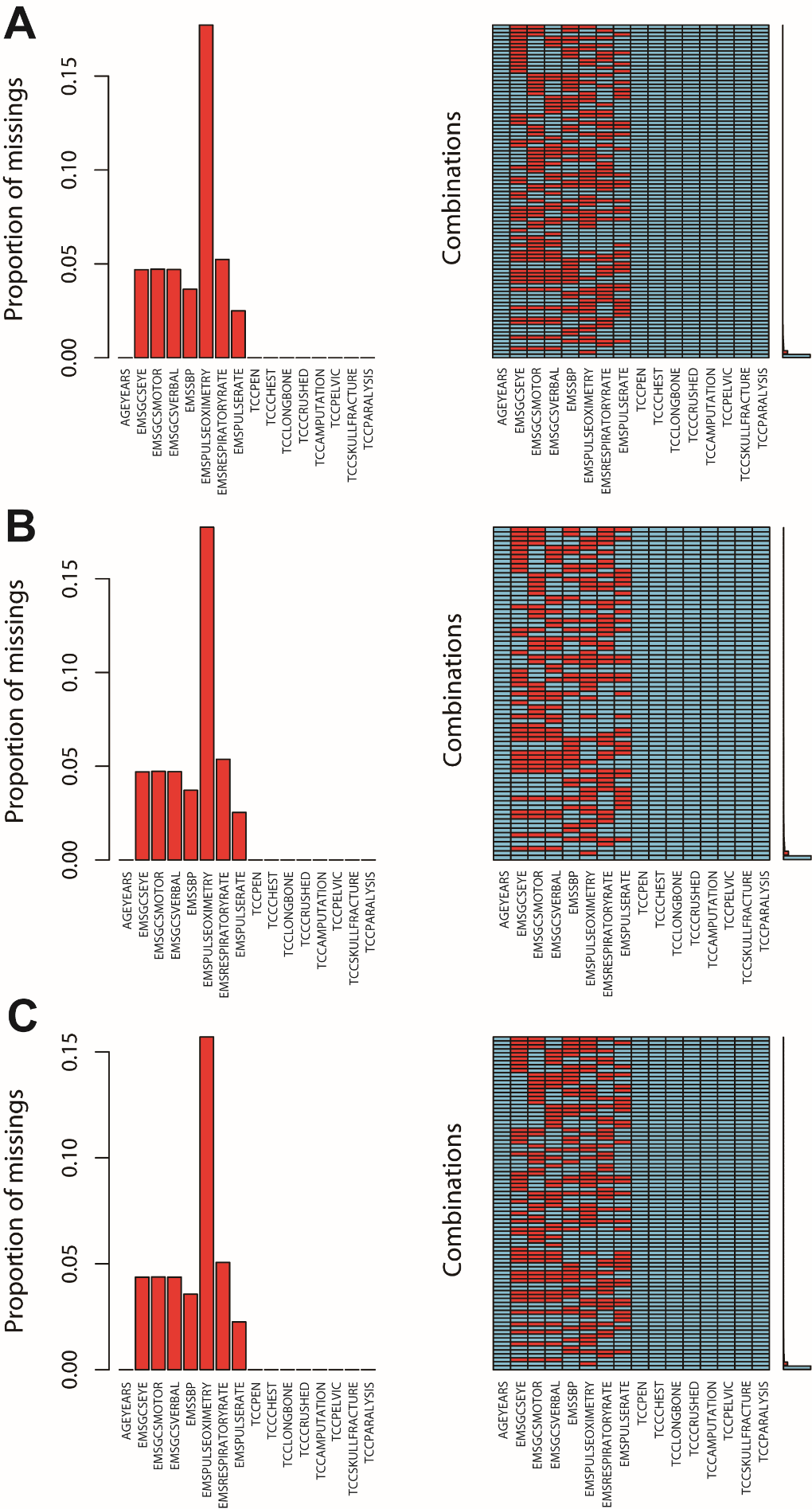

Supplement: Multimedia Appendix 4 [file jmir_v26i1e58740_app4.docx]

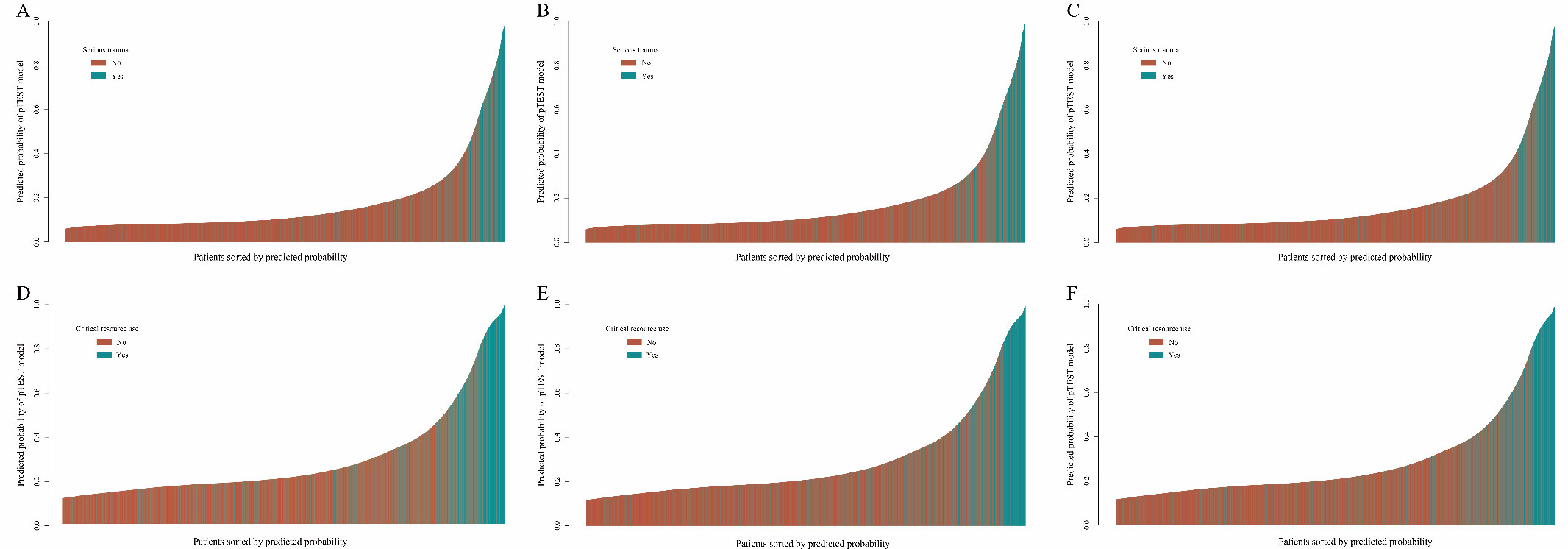

Supplement: Multimedia Appendix 14 [file jmir_v26i1e58740_app14.docx]
