# Supplementary material for: Enhancing Performance of the National Field Triage Guidelines Using Machine Learning: Development of a Prehospital Triage Model to Predict Severe Trauma
Source: J Med Internet Res. 2024 Sep 30;26:e58740. doi: 10.2196/58740 (PMC11474124; doi:10.2196/58740)
Supplement: Multimedia Appendix 5 [file jmir_v26i1e58740_app5.docx]

| **Characteristics** | **Non-severe trauma (n=552619)** | **severe trauma (n=119690)** | ***P* value** |
| --- | --- | --- | --- |
| Sex |  |  |  |
| * Male | 319626(57.85) | 83808(70.03) | <.001 |
| * Female | 232923(42.15) | 35865(29.97) |  |
| * Total | 552549(100.00) | 119673(100.00) |  |
| Transport mode |  |  |  |
| * Ground | 523225(94.68) | 99264(82.93) | <.001 |
| * Helicopter | 28573(5.17) | 19891(16.62) |  |
| * Fixed-wing | 821(0.15) | 535(0.45) |  |
| * Total | 552619(100.00) | 119690(100.00) |  |
| Trauma center level |  |  |  |
| * Level 1 | 218075(54.00) | 57648(63.57) | <.001 |
| * Level 2 | 149305(36.97) | 30114(33.21) |  |
| * Level 3 | 36469(9.03) | 2928(3.23) |  |
| * Total | 403849(100.00) | 90690(100.00) |  |
| TCCPEN |  |  |  |
| * No | 531763(96.23) | 113559(94.88) | <.001 |
| * Yes | 20856(3.77) | 6131(5.12) |  |
| * Total | 552619(100.00) | 119690(100.00) |  |
| TCCCHEST |  |  |  |
| * No | 551957(99.88) | 116057(96.96) | <.001 |
| * Yes | 662(0.12) | 3633(3.04) |  |
| * Total | 552619(100.00) | 119690(100.00) |  |
| TCCLONGBONE |  |  |  |
| * No | 549797(99.49) | 117914(98.52) | <.001 |
| * Yes | 2822(0.51) | 1776(1.48) |  |
| * Total | 552619(100.00) | 119690(100.00) |  |
| TCCCRUSHED |  |  |  |
| * No | 550320(99.58) | 118801(99.26) | <.001 |
| * Yes | 2299(0.42) | 889(0.74) |  |
| * Total | 552619(100.00) | 119690(100.00) |  |
| TCCAMPUTATION |  |  |  |
| * No | 552243(99.93) | 119234(99.62) | <.001 |
| * Yes | 376(0.07) | 456(0.38) |  |
| * Total | 552619(100.00) | 119690(100.00) |  |
| TCCPELVIC |  |  |  |
| * No | 550527(99.62) | 114287(95.49) | <.001 |
| * Yes | 2092(0.38) | 5403(4.51) |  |
| * Total | 552619(100.00) | 119690(100.00) |  |
| TCCSKULLFRACTURE |  |  |  |
| * No | 551992(99.89) | 115190(96.24) | <.001 |
| * Yes | 627(0.11) | 4500(3.76) |  |
| * Total | 552619(100.00) | 119690(100.00) |  |
| TCCPARALYSIS |  |  |  |
| * No | 551675(99.83) | 116255(97.13) | <.001 |
| * Yes | 944(0.17) | 3435(2.87) |  |
| * Total | 552619(100.00) | 119690(100.00) |  |
| Surgery for hemorrhage control |  |  |  |
| * No | 483645(99.18) | 102958(90.57) | <.001 |
| * Yes | 3996(0.82) | 10718(9.43) |  |
| * Total | 487641(100.00) | 113676(100.00) |  |
| Cerebral monitor |  |  |  |
| * No | 487290(99.85) | 105957(93.15) | <.001 |
| * Yes | 738(0.15) | 7789(6.85) |  |
| * Total | 488028(100.00) | 113746(100.00) |  |
| Interventional radiology procedures |  |  |  |
| * No | 486623(99.80) | 109398(96.32) | <.001 |
| * Yes | 977(0.20) | 4177(3.68) |  |
| * Total | 487600(100.00) | 113575(100.00) |  |
| Discharge to the ICU from ED |  |  |  |
| * No | 467620(85.74) | 53567(45.32) | <.001 |
| * Yes | 77804(14.26) | 64618(54.68) |  |
| * Total | 545424(100.00) | 118185(100.00) |  |
| In-hospital death within 24 h |  |  |  |
| * No | 551016(99.72) | 111895(93.51) | <.001 |
| * Yes | 1543(0.28) | 7760(6.49) |  |
| * Total | 552559(100.00) | 119655(100.00) |  |
| Intubation in the EMS or ED |  |  |  |
| * No | 524031(94.83) | 75523(63.10) | <.001 |
| * Yes | 28588(5.17) | 44167(36.90) |  |
| * Total | 552619(100.00) | 119690(100.00) |  |
| Critical resource use |  |  |  |
| * No | 396358(81.20) | 30878(26.46) | <.001 |
| * Yes | 91769(18.80) | 85801(73.54) |  |
| * Total | 488127(100.00) | 116679(100.00) |  |
| RED criteria |  |  |  |
| * No | 502090(90.86) | 82642(69.05) | <.001 |
| * Yes | 50529(9.14) | 37048(30.95) |  |
| * Total | 552619(100.00) | 119690(100.00) |  |
| Age |  |  |  |
| * N(Missing) | 552619(0) | 119690(0) | <.001 |
| * Mean(SD) | 53.79(21.96) | 50.01(21.12) |  |
| * Median | 55 | 50 |  |
| * Q1,Q3 | 34.00,73.00 | 31.00,67.00 |  |
| EMSSBP |  |  |  |
| * N(Missing) | 535189(17430) | 112578(7112) | <.001 |
| * Mean(SD) | 141.06(27.36) | 134.33(32.13) |  |
| * Median | 140 | 134 |  |
| * Q1,Q3 | 124.00,157.00 | 113.00,153.00 |  |
| EMSPULSERATE |  |  |  |
| * N(Missing) | 539493(13126) | 115991(3699) | <.001 |
| * Mean(SD) | 90.07(19.58) | 92.95(23.26) |  |
| * Median | 88 | 90 |  |
| * Q1,Q3 | 77.00,101.00 | 77.00,108.00 |  |
| EMSRESPIRATORYRATE |  |  |  |
| * N(Missing) | 524177(28442) | 112948(6742) | <.001 |
| * Mean(SD) | 18.30(4.35) | 18.97(6.18) |  |
| * Median | 18 | 18 |  |
| * Q1,Q3 | 16.00,20.00 | 16.00,20.00 |  |
| EMSPULSEOXIMETRY |  |  |  |
| * N(Missing) | 455692(96927) | 97527(22163) | <.001 |
| * Mean(SD) | 96.56(4.96) | 94.83(7.35) |  |
| * Median | 98 | 97 |  |
| * Q1,Q3 | 96.00,99.00 | 94.00,98.00 |  |
| EMSGCSEYE |  |  |  |
| * N(Missing) | 527928(24691) | 112806(6884) | <.001 |
| * Mean(SD) | 3.90(0.45) | 3.37(1.12) |  |
| * Median | 4 | 4 |  |
| * Q1,Q3 | 4.00,4.00 | 3.00,4.00 |  |
| EMSGCSVERBAL |  |  |  |
| * N(Missing) | 527928(24691) | 112798(6892) | <.001 |
| * Mean(SD) | 4.75(0.71) | 3.93(1.50) |  |
| * Median | 5 | 5 |  |
| * Q1,Q3 | 5.00,5.00 | 4.00,5.00 |  |
| EMSGCSMOTOR |  |  |  |
| * N(Missing) | 527827(24792) | 112755(6935) | <.001 |
| * Mean(SD) | 5.87(0.64) | 5.08(1.71) |  |
| * Median | 6 | 6 |  |
| * Q1,Q3 | 6.00,6.00 | 5.00,6.00 |  |
| EMSTOTALGCS |  |  |  |
| * N(Missing) | 532573(20046) | 115141(4549) | <.001 |
| * Mean(SD) | 14.51(1.65) | 12.35(4.12) |  |
| * Median | 15 | 15 |  |
| * Q1,Q3 | 15.00,15.00 | 12.00,15.00 |  |
| Minutes spent in ED |  |  |  |
| * N(Missing) | 527293(25326) | 113484(6206) | <.001 |
| * Mean(SD) | 201.04(152.36) | 138.37(128.66) |  |
| * Median | 162 | 101 |  |
| * Q1,Q3 | 97.00,262.00 | 53.00,180.00 |  |
| Length of stay (days) |  |  |  |
| * N(Missing) | 545670(6949) | 117120(2570) | <.001 |
| * Mean(SD) | 5.01(7.82) | 11.77(14.36) |  |
| * Median | 4 | 8 |  |
| * Q1,Q3 | 2.00,6.00 | 4.00,14.00 |  |
| ISS score |  |  |  |
| * N(Missing) | 552619(0) | 119690(0) | <.001 |
| * Mean(SD) | 6.58(3.74) | 24.02(8.92) |  |
| * Median | 5 | 22 |  |
| * Q1,Q3 | 4.00,9.00 | 17.00,27.00 |  |
| PHI score |  |  |  |
| * N(Missing) | 494830(57789) | 102860(16830) | <.001 |
| * Mean(SD) | 1.01(1.85) | 2.79(3.15) |  |
| * Median | 0 | 3 |  |
| * Q1,Q3 | 0.00,3.00 | 0.00,5.00 |  |
| RTS score |  |  |  |
| * N(Missing) | 496610(56009) | 103259(16431) | <.001 |
| * Mean(SD) | 11.86(0.58) | 11.14(1.57) |  |
| * Median | 12 | 12 |  |
| * Q1,Q3 | 12.00,12.00 | 11.00,12.00 |  |
