# Supplementary material for: Enhancing Performance of the National Field Triage Guidelines Using Machine Learning: Development of a Prehospital Triage Model to Predict Severe Trauma
Source: J Med Internet Res. 2024 Sep 30;26:e58740. doi: 10.2196/58740 (PMC11474124; doi:10.2196/58740)
Supplement: Multimedia Appendix 6 [file jmir_v26i1e58740_app6.docx]

| **Characteristics** | **Non-severe trauma (n=236838)** | **Severe trauma (n=51296)** | ***P* value** |
| --- | --- | --- | --- |
| Sex |  |  |  |
| * Male | 136888(57.80) | 35933(70.06) | <.001 |
| * Female | 99939(42.20) | 15358(29.94) |  |
| * Total | 236827(100.00) | 51291(100.00) |  |
| Transport mode |  |  |  |
| * Ground | 224143(94.64) | 42479(82.81) | <.001 |
| * Helicopter | 12327(5.20) | 8584(16.73) |  |
| * Fixed-wing | 368(0.16) | 233(0.45) |  |
| * Total | 236838(100.00) | 51296(100.00) |  |
| Trauma center level |  |  |  |
| * Level 1 | 93523(53.96) | 24676(63.53) | <.001 |
| * Level 2 | 64068(36.97) | 12937(33.31) |  |
| * Level 3 | 15718(9.07) | 1226(3.16) |  |
| * Total | 173309(100.00) | 38839(100.00) |  |
| TCCPEN |  |  |  |
| * No | 228059(96.29) | 48756(95.05) | <.001 |
| * Yes | 8779(3.71) | 2540(4.95) |  |
| * Total | 236838(100.00) | 51296(100.00) |  |
| TCCCHEST |  |  |  |
| * No | 236556(99.88) | 49753(96.99) | <.001 |
| * Yes | 282(0.12) | 1543(3.01) |  |
| * Total | 236838(100.00) | 51296(100.00) |  |
| TCCLONGBONE |  |  |  |
| * No | 235682(99.51) | 50585(98.61) | <.001 |
| * Yes | 1156(0.49) | 711(1.39) |  |
| * Total | 236838(100.00) | 51296(100.00) |  |
| TCCCRUSHED |  |  |  |
| * No | 235856(99.59) | 50904(99.24) | <.001 |
| * Yes | 982(0.41) | 392(0.76) |  |
| * Total | 236838(100.00) | 51296(100.00) |  |
| TCCAMPUTATION |  |  |  |
| * No | 236682(99.93) | 51080(99.58) | <.001 |
| * Yes | 156(0.07) | 216(0.42) |  |
| * Total | 236838(100.00) | 51296(100.00) |  |
| TCCPELVIC |  |  |  |
| * No | 235936(99.62) | 48852(95.24) | <.001 |
| * Yes | 902(0.38) | 2444(4.76) |  |
| * Total | 236838(100.00) | 51296(100.00) |  |
| TCCSKULLFRACTURE |  |  |  |
| * No | 236538(99.87) | 49359(96.22) | <.001 |
| * Yes | 300(0.13) | 1937(3.78) |  |
| * Total | 236838(100.00) | 51296(100.00) |  |
| TCCPARALYSIS |  |  |  |
| * No | 236435(99.83) | 49812(97.11) | <.001 |
| * Yes | 403(0.17) | 1484(2.89) |  |
| * Total | 236838(100.00) | 51296(100.00) |  |
| Surgery for hemorrhage control |  |  |  |
| * No | 207124(99.16) | 44019(90.37) | <.001 |
| * Yes | 1751(0.84) | 4690(9.63) |  |
| * Total | 208875(100.00) | 48709(100.00) |  |
| Cerebral monitor |  |  |  |
| * No | 208765(99.86) | 45463(93.29) | <.001 |
| * Yes | 294(0.14) | 3269(6.71) |  |
| * Total | 209059(100.00) | 48732(100.00) |  |
| Interventional radiology procedures |  |  |  |
| * No | 208480(99.82) | 46862(96.30) | <.001 |
| * Yes | 382(0.18) | 1800(3.70) |  |
| * Total | 208862(100.00) | 48662(100.00) |  |
| Discharge to the ICU from ED |  |  |  |
| * No | 200216(85.64) | 22820(45.07) | <.001 |
| * Yes | 33571(14.36) | 27813(54.93) |  |
| * Total | 233787(100.00) | 50633(100.00) |  |
| In-hospital death within 24 h |  |  |  |
| * No | 236145(99.71) | 47975(93.55) | <.001 |
| * Yes | 678(0.29) | 3309(6.45) |  |
| * Total | 236823(100.00) | 51284(100.00) |  |
| Intubation in the EMS or ED |  |  |  |
| * No | 224602(94.83) | 32452(63.26) | <.001 |
| * Yes | 12236(5.17) | 18844(36.74) |  |
| * Total | 236838(100.00) | 51296(100.00) |  |
| Critical resource use |  |  |  |
| * No | 169448(81.01) | 13096(26.20) | <.001 |
| * Yes | 39723(18.99) | 36881(73.80) |  |
| * Total | 209171(100.00) | 49977(100.00) |  |
| RED criteria |  |  |  |
| * No | 215273(90.89) | 35413(69.04) | <.001 |
| * Yes | 21565(9.11) | 15883(30.96) |  |
| * Total | 236838(100.00) | 51296(100.00) |  |
| Age |  |  |  |
| * N(Missing) | 236838(0) | 51296(0) | <.001 |
| * Mean(SD) | 53.91(21.93) | 50.01(21.15) |  |
| * Median | 56 | 50 |  |
| * Q1,Q3 | 34.00,73.00 | 31.00,67.00 |  |
| EMSSBP |  |  |  |
| * N(Missing) | 229187(7651) | 48241(3055) | <.001 |
| * Mean(SD) | 141.13(27.40) | 134.16(32.22) |  |
| * Median | 140 | 133 |  |
| * Q1,Q3 | 124.00,158.00 | 113.00,153.00 |  |
| EMSPULSERATE |  |  |  |
| * N(Missing) | 231164(5674) | 49676(1620) | <.001 |
| * Mean(SD) | 90.09(19.53) | 92.88(23.38) |  |
| * Median | 88 | 90 |  |
| * Q1,Q3 | 77.00,101.00 | 77.00,108.00 |  |
| EMSRESPIRATORYRATE |  |  |  |
| * N(Missing) | 224349(12489) | 48310(2986) | <.001 |
| * Mean(SD) | 18.31(4.39) | 19.01(6.31) |  |
| * Median | 18 | 18 |  |
| * Q1,Q3 | 16.00,20.00 | 16.00,20.00 |  |
| EMSPULSEOXIMETRY |  |  |  |
| * N(Missing) | 195243(41595) | 41761(9535) | <.001 |
| * Mean(SD) | 96.55(4.98) | 94.89(7.20) |  |
| * Median | 98 | 97 |  |
| * Q1,Q3 | 96.00,99.00 | 94.00,98.00 |  |
| EMSGCSEYE |  |  |  |
| * N(Missing) | 226191(10647) | 48405(2891) | <.001 |
| * Mean(SD) | 3.90(0.44) | 3.37(1.12) |  |
| * Median | 4 | 4 |  |
| * Q1,Q3 | 4.00,4.00 | 3.00,4.00 |  |
| EMSGCSVERBAL |  |  |  |
| * N(Missing) | 226170(10668) | 48405(2891) | <.001 |
| * Mean(SD) | 4.75(0.71) | 3.93(1.49) |  |
| * Median | 5 | 5 |  |
| * Q1,Q3 | 5.00,5.00 | 4.00,5.00 |  |
| EMSGCSMOTOR |  |  |  |
| * N(Missing) | 226142(10696) | 48375(2921) | <.001 |
| * Mean(SD) | 5.87(0.64) | 5.09(1.71) |  |
| * Median | 6 | 6 |  |
| * Q1,Q3 | 6.00,6.00 | 5.00,6.00 |  |
| EMSTOTALGCS |  |  |  |
| * N(Missing) | 228305(8533) | 49386(1910) | <.001 |
| * Mean(SD) | 14.50(1.65) | 12.36(4.11) |  |
| * Median | 15 | 15 |  |
| * Q1,Q3 | 15.00,15.00 | 12.00,15.00 |  |
| Minutes spent in ED |  |  |  |
| * N(Missing) | 226133(10705) | 48669(2627) | <.001 |
| * Mean(SD) | 219.74(414.13) | 152.00(325.63) |  |
| * Median | 165 | 100 |  |
| * Q1,Q3 | 97.00,268.00 | 53.00,182.00 |  |
| Length of stay (days) |  |  |  |
| * N(Missing) | 233887(2951) | 50207(1089) | <.001 |
| * Mean(SD) | 5.02(6.05) | 11.75(14.23) |  |
| * Median | 4 | 8 |  |
| * Q1,Q3 | 2.00,6.00 | 4.00,14.00 |  |
| ISS score |  |  |  |
| * N(Missing) | 236838(0) | 51296(0) | <.001 |
| * Mean(SD) | 6.60(3.73) | 23.95(8.79) |  |
| * Median | 5 | 22 |  |
| * Q1,Q3 | 4.00,9.00 | 17.00,27.00 |  |
| PHI score |  |  |  |
| * N(Missing) | 211816(25022) | 44042(7254) | <.001 |
| * Mean(SD) | 1.01(1.84) | 2.79(3.15) |  |
| * Median | 0 | 3 |  |
| * Q1,Q3 | 0.00,3.00 | 0.00,5.00 |  |
| RTS score |  |  |  |
| * N(Missing) | 212509(24329) | 44248(7048) | <.001 |
| * Mean(SD) | 11.85(0.59) | 11.14(1.57) |  |
| * Median | 12 | 12 |  |
| * Q1,Q3 | 12.00,12.00 | 11.00,12.00 |  |
