# Supplementary material for: Enhancing Performance of the National Field Triage Guidelines Using Machine Learning: Development of a Prehospital Triage Model to Predict Severe Trauma
Source: J Med Internet Res. 2024 Sep 30;26:e58740. doi: 10.2196/58740 (PMC11474124; doi:10.2196/58740)
Supplement: Multimedia Appendix 7 [file jmir_v26i1e58740_app7.docx]

| **Characteristics** | **Non-severe trauma (n=421801)** | **Severe trauma (n=86902)** | ***P* value** |
| --- | --- | --- | --- |
| Sex |  |  |  |
| * Male | 240214(56.96) | 60497(69.63) | <.001 |
| * Female | 181533(43.04) | 26388(30.37) |  |
| * Total | 421747(100.00) | 86885(100.00) |  |
| Transport mode |  |  |  |
| * Ground | 401705(95.24) | 72940(83.93) | <.001 |
| * Helicopter | 19651(4.66) | 13695(15.76) |  |
| * Fixed-wing | 445(0.11) | 267(0.31) |  |
| * Total | 421801(100.00) | 86902(100.00) |  |
| Trauma center level |  |  |  |
| * Level 1 | 164513(52.54) | 42050(63.02) | <.001 |
| * Level 2 | 120036(38.34) | 22397(33.57) |  |
| * Level 3 | 28542(9.12) | 2277(3.41) |  |
| * Total | 313091(100.00) | 66724(100.00) |  |
| TCCPEN |  |  |  |
| * No | 408108(96.75) | 82937(95.44) | <.001 |
| * Yes | 13693(3.25) | 3965(4.56) |  |
| * Total | 421801(100.00) | 86902(100.00) |  |
| TCCCHEST |  |  |  |
| * No | 421242(99.87) | 84245(96.94) | <.001 |
| * Yes | 559(0.13) | 2657(3.06) |  |
| * Total | 421801(100.00) | 86902(100.00) |  |
| TCCLONGBONE |  |  |  |
| * No | 419868(99.54) | 85622(98.53) | <.001 |
| * Yes | 1933(0.46) | 1280(1.47) |  |
| * Total | 421801(100.00) | 86902(100.00) |  |
| TCCCRUSHED |  |  |  |
| * No | 419861(99.54) | 86130(99.11) | <.001 |
| * Yes | 1940(0.46) | 772(0.89) |  |
| * Total | 421801(100.00) | 86902(100.00) |  |
| TCCAMPUTATION |  |  |  |
| * No | 421502(99.93) | 86588(99.64) | <.001 |
| * Yes | 299(0.07) | 314(0.36) |  |
| * Total | 421801(100.00) | 86902(100.00) |  |
| TCCPELVIC |  |  |  |
| * No | 420069(99.59) | 82782(95.26) | <.001 |
| * Yes | 1732(0.41) | 4120(4.74) |  |
| * Total | 421801(100.00) | 86902(100.00) |  |
| TCCSKULLFRACTURE |  |  |  |
| * No | 421074(99.83) | 83542(96.13) | <.001 |
| * Yes | 727(0.17) | 3360(3.87) |  |
| * Total | 421801(100.00) | 86902(100.00) |  |
| TCCPARALYSIS |  |  |  |
| * No | 421134(99.84) | 84492(97.23) | <.001 |
| * Yes | 667(0.16) | 2410(2.77) |  |
| * Total | 421801(100.00) | 86902(100.00) |  |
| Surgery for hemorrhage control |  |  |  |
| * No | 372865(99.19) | 75035(90.53) | <.001 |
| * Yes | 3038(0.81) | 7848(9.47) |  |
| * Total | 375903(100.00) | 82883(100.00) |  |
| Cerebral monitor |  |  |  |
| * No | 376102(99.88) | 77662(93.54) | <.001 |
| * Yes | 457(0.12) | 5362(6.46) |  |
| * Total | 376559(100.00) | 83024(100.00) |  |
| Interventional radiology procedures |  |  |  |
| * No | 375158(99.80) | 79756(96.25) | <.001 |
| * Yes | 742(0.20) | 3106(3.75) |  |
| * Total | 375900(100.00) | 82862(100.00) |  |
| Discharge to the ICU from ED |  |  |  |
| * No | 357845(86.03) | 38180(44.53) | <.001 |
| * Yes | 58115(13.97) | 47555(55.47) |  |
| * Total | 415960(100.00) | 85735(100.00) |  |
| In-hospital death within 24 h |  |  |  |
| * No | 420697(99.74) | 81635(93.94) | <.001 |
| * Yes | 1104(0.26) | 5267(6.06) |  |
| * Total | 421801(100.00) | 86902(100.00) |  |
| Intubation in the EMS or ED |  |  |  |
| * No | 402733(95.48) | 56191(64.66) | <.001 |
| * Yes | 19068(4.52) | 30711(35.34) |  |
| * Total | 421801(100.00) | 86902(100.00) |  |
| Critical resource use |  |  |  |
| * No | 308220(82.14) | 22072(26.09) | <.001 |
| * Yes | 67035(17.86) | 62516(73.91) |  |
| * Total | 375255(100.00) | 84588(100.00) |  |
| RED criteria |  |  |  |
| * No | 385521(91.40) | 60988(70.18) | <.001 |
| * Yes | 36280(8.60) | 25914(29.82) |  |
| * Total | 421801(100.00) | 86902(100.00) |  |
| Age |  |  |  |
| * N(Missing) | 421801(0) | 86902(0) | <.001 |
| * Mean(SD) | 55.10(21.84) | 51.00(21.10) |  |
| * Median | 58 | 52 |  |
| * Q1,Q3 | 35.00,74.00 | 32.00,69.00 |  |
| EMSSBP |  |  |  |
| * N(Missing) | 408858(12943) | 81707(5195) | <.001 |
| * Mean(SD) | 141.91(27.62) | 134.87(32.54) |  |
| * Median | 140 | 134 |  |
| * Q1,Q3 | 124.00,158.00 | 114.00,154.00 |  |
| EMSPULSERATE |  |  |  |
| * N(Missing) | 412873(8928) | 84360(2542) | <.001 |
| * Mean(SD) | 89.86(19.73) | 92.72(23.33) |  |
| * Median | 88 | 90 |  |
| * Q1,Q3 | 76.00,101.00 | 77.00,107.00 |  |
| EMSRESPIRATORYRATE |  |  |  |
| * N(Missing) | 400925(20876) | 82020(4882) | <.001 |
| * Mean(SD) | 18.34(4.45) | 19.06(6.29) |  |
| * Median | 18 | 18 |  |
| * Q1,Q3 | 16.00,20.00 | 16.00,21.00 |  |
| EMSPULSEOXIMETRY |  |  |  |
| * N(Missing) | 356696(65105) | 72111(14791) | <.001 |
| * Mean(SD) | 96.49(4.91) | 94.73(7.36) |  |
| * Median | 98 | 97 |  |
| * Q1,Q3 | 96.00,99.00 | 94.00,98.00 |  |
| EMSGCSEYE |  |  |  |
| * N(Missing) | 404002(17799) | 82489(4413) | <.001 |
| * Mean(SD) | 3.91(0.44) | 3.39(1.11) |  |
| * Median | 4 | 4 |  |
| * Q1,Q3 | 4.00,4.00 | 3.00,4.00 |  |
| EMSGCSVERBAL |  |  |  |
| * N(Missing) | 403981(17820) | 82493(4409) | <.001 |
| * Mean(SD) | 4.75(0.70) | 3.95(1.47) |  |
| * Median | 5 | 5 |  |
| * Q1,Q3 | 5.00,5.00 | 4.00,5.00 |  |
| EMSGCSMOTOR |  |  |  |
| * N(Missing) | 403959(17842) | 82465(4437) | <.001 |
| * Mean(SD) | 5.88(0.62) | 5.11(1.68) |  |
| * Median | 6 | 6 |  |
| * Q1,Q3 | 6.00,6.00 | 5.00,6.00 |  |
| EMSTOTALGCS |  |  |  |
| * N(Missing) | 405350(16451) | 83110(3792) | <.001 |
| * Mean(SD) | 14.52(1.62) | 12.44(4.06) |  |
| * Median | 15 | 15 |  |
| * Q1,Q3 | 15.00,15.00 | 12.00,15.00 |  |
| Minutes spent in ED |  |  |  |
| * N(Missing) | 405933(15868) | 83186(3716) | <.001 |
| * Mean(SD) | 220.46(1478.17) | 145.34(359.18) |  |
| * Median | 163.2 | 97.8 |  |
| * Q1,Q3 | 97.20,265.20 | 51.00,175.20 |  |
| Length of stay (days) |  |  |  |
| * N(Missing) | 416979(4822) | 85508(1394) | <.001 |
| * Mean(SD) | 5.04(5.83) | 11.91(14.41) |  |
| * Median | 4 | 8 |  |
| * Q1,Q3 | 2.00,6.00 | 4.00,14.00 |  |
| ISS score |  |  |  |
| * N(Missing) | 421801(0) | 86902(0) | <.001 |
| * Mean(SD) | 6.59(3.72) | 23.94(8.75) |  |
| * Median | 5 | 22 |  |
| * Q1,Q3 | 4.00,9.00 | 17.00,27.00 |  |
| PHI score |  |  |  |
| * N(Missing) | 379569(42232) | 75135(11767) | <.001 |
| * Mean(SD) | 1.02(1.86) | 2.77(3.14) |  |
| * Median | 0 | 3 |  |
| * Q1,Q3 | 0.00,3.00 | 0.00,5.00 |  |
| RTS score |  |  |  |
| * N(Missing) | 380790(41011) | 75427(11475) | <.001 |
| * Mean(SD) | 11.85(0.58) | 11.16(1.54) |  |
| * Median | 12 | 12 |  |
| * Q1,Q3 | 12.00,12.00 | 11.00,12.00 |  |
