# Supplementary material for: Enhancing Performance of the National Field Triage Guidelines Using Machine Learning: Development of a Prehospital Triage Model to Predict Severe Trauma
Source: J Med Internet Res. 2024 Sep 30;26:e58740. doi: 10.2196/58740 (PMC11474124; doi:10.2196/58740)
Supplement: Multimedia Appendix 8 [file jmir_v26i1e58740_app8.docx]

| **Characteristics** | **No critical resource use (n=427236)** | **Critical resource use (n=177570)** | ***P* value** |
| --- | --- | --- | --- |
| Sex |  |  |  |
| * Male | 248208(58.10) | 121665(68.53) | <.001 |
| * Female | 178974(41.90) | 55878(31.47) |  |
| * Total | 427182(100.00) | 177543(100.00) |  |
| Transport mode |  |  |  |
| * Ground | 404057(94.57) | 152276(85.76) | <.001 |
| * Helicopter | 22520(5.27) | 24683(13.90) |  |
| * Fixed-wing | 659(0.15) | 611(0.34) |  |
| * Total | 427236(100.00) | 177570(100.00) |  |
| Trauma center level |  |  |  |
| * Level 1 | 192520(58.33) | 79976(60.30) | <.001 |
| * Level 2 | 127987(38.78) | 47497(35.81) |  |
| * Level 3 | 9540(2.89) | 5153(3.89) |  |
| * Total | 330047(100.00) | 132626(100.00) |  |
| TCCPEN |  |  |  |
| * No | 411212(96.25) | 168403(94.84) | <.001 |
| * Yes | 16024(3.75) | 9167(5.16) |  |
| * Total | 427236(100.00) | 177570(100.00) |  |
| TCCCHEST |  |  |  |
| * No | 426267(99.77) | 174347(98.18) | <.001 |
| * Yes | 969(0.23) | 3223(1.82) |  |
| * Total | 427236(100.00) | 177570(100.00) |  |
| TCCLONGBONE |  |  |  |
| * No | 424665(99.40) | 175766(98.98) | <.001 |
| * Yes | 2571(0.60) | 1804(1.02) |  |
| * Total | 427236(100.00) | 177570(100.00) |  |
| TCCCRUSHED |  |  |  |
| * No | 425336(99.56) | 176430(99.36) | <.001 |
| * Yes | 1900(0.44) | 1140(0.64) |  |
| * Total | 427236(100.00) | 177570(100.00) |  |
| TCCAMPUTATION |  |  |  |
| * No | 426917(99.93) | 177081(99.72) | <.001 |
| * Yes | 319(0.07) | 489(0.28) |  |
| * Total | 427236(100.00) | 177570(100.00) |  |
| TCCPELVIC |  |  |  |
| * No | 424393(99.33) | 173228(97.55) | <.001 |
| * Yes | 2843(0.67) | 4342(2.45) |  |
| * Total | 427236(100.00) | 177570(100.00) |  |
| TCCSKULLFRACTURE |  |  |  |
| * No | 426383(99.80) | 173379(97.64) | <.001 |
| * Yes | 853(0.20) | 4191(2.36) |  |
| * Total | 427236(100.00) | 177570(100.00) |  |
| TCCPARALYSIS |  |  |  |
| * No | 426149(99.75) | 174375(98.20) | <.001 |
| * Yes | 1087(0.25) | 3195(1.80) |  |
| * Total | 427236(100.00) | 177570(100.00) |  |
| ISS score |  |  |  |
| * <16 | 396358(92.77) | 91769(51.68) | <.001 |
| * >=16 | 30878(7.23) | 85801(48.32) |  |
| * Total | 427236(100.00) | 177570(100.00) |  |
| RED criteria |  |  |  |
| * No | 389530(91.17) | 131637(74.13) | <.001 |
| * Yes | 37706(8.83) | 45933(25.87) |  |
| * Total | 427236(100.00) | 177570(100.00) |  |
| Age |  |  |  |
| * N(Missing) | 427236(0) | 177570(0) | <.001 |
| * Mean(SD) | 52.67(21.96) | 52.05(21.32) |  |
| * Median | 54 | 53 |  |
| * Q1,Q3 | 32.00,72.00 | 32.00,70.00 |  |
| EMSSBP |  |  |  |
| * N(Missing) | 414673(12563) | 167545(10025) | <.001 |
| * Mean(SD) | 140.83(26.80) | 136.31(32.13) |  |
| * Median | 140 | 136 |  |
| * Q1,Q3 | 124.00,157.00 | 116.00,155.00 |  |
| EMSPULSERATE |  |  |  |
| * N(Missing) | 417173(10063) | 172177(5393) | <.001 |
| * Mean(SD) | 89.87(19.10) | 93.20(23.27) |  |
| * Median | 88 | 90 |  |
| * Q1,Q3 | 77.00,100.00 | 78.00,108.00 |  |
| EMSRESPIRATORYRATE |  |  |  |
| * N(Missing) | 406220(21016) | 167617(9953) | <.001 |
| * Mean(SD) | 18.30(4.20) | 18.83(5.97) |  |
| * Median | 18 | 18 |  |
| * Q1,Q3 | 16.00,20.00 | 16.00,20.00 |  |
| EMSPULSEOXIMETRY |  |  |  |
| * N(Missing) | 351437(75799) | 143730(33840) | <.001 |
| * Mean(SD) | 96.73(4.73) | 95.05(7.12) |  |
| * Median | 98 | 97 |  |
| * Q1,Q3 | 96.00,99.00 | 94.00,98.00 |  |
| EMSGCSEYE |  |  |  |
| * N(Missing) | 408941(18295) | 167482(10088) | <.001 |
| * Mean(SD) | 3.94(0.32) | 3.43(1.08) |  |
| * Median | 4 | 4 |  |
| * Q1,Q3 | 4.00,4.00 | 3.00,4.00 |  |
| EMSGCSVERBAL |  |  |  |
| * N(Missing) | 408918(18318) | 167489(10081) | <.001 |
| * Mean(SD) | 4.81(0.55) | 3.99(1.46) |  |
| * Median | 5 | 5 |  |
| * Q1,Q3 | 5.00,5.00 | 4.00,5.00 |  |
| EMSGCSMOTOR |  |  |  |
| * N(Missing) | 408845(18391) | 167426(10144) | <.001 |
| * Mean(SD) | 5.93(0.42) | 5.16(1.63) |  |
| * Median | 6 | 6 |  |
| * Q1,Q3 | 6.00,6.00 | 5.00,6.00 |  |
| EMSTOTALGCS |  |  |  |
| * N(Missing) | 412154(15082) | 170866(6704) | <.001 |
| * Mean(SD) | 14.68(1.14) | 12.55(3.95) |  |
| * Median | 15 | 15 |  |
| * Q1,Q3 | 15.00,15.00 | 12.00,15.00 |  |
| Minutes spent in ED |  |  |  |
| * N(Missing) | 413369(13867) | 170368(7202) | <.001 |
| * Mean(SD) | 210.20(156.91) | 137.17(124.67) |  |
| * Median | 170 | 101 |  |
| * Q1,Q3 | 102.00,274.00 | 53.00,179.00 |  |
| Length of stay (days) |  |  |  |
| * N(Missing) | 421992(5244) | 174075(3495) | <.001 |
| * Mean(SD) | 4.74(7.80) | 10.42(13.07) |  |
| * Median | 4 | 7 |  |
| * Q1,Q3 | 2.00,6.00 | 4.00,12.00 |  |
| ISS score |  |  |  |
| * N(Missing) | 427236(0) | 177570(0) | <.001 |
| * Mean(SD) | 7.29(5.23) | 16.64(11.19) |  |
| * Median | 5 | 14 |  |
| * Q1,Q3 | 4.00,9.00 | 9.00,22.00 |  |
| PHI score |  |  |  |
| * N(Missing) | 384933(42303) | 153059(24511) | <.001 |
| * Mean(SD) | 0.89(1.67) | 2.64(3.04) |  |
| * Median | 0 | 3 |  |
| * Q1,Q3 | 0.00,1.00 | 0.00,4.00 |  |
| RTS score |  |  |  |
| * N(Missing) | 386253(40983) | 153645(23925) | <.001 |
| * Mean(SD) | 11.91(0.42) | 11.22(1.48) |  |
| * Median | 12 | 12 |  |
| * Q1,Q3 | 12.00,12.00 | 11.00,12.00 |  |
