# Supplementary material for: Enhancing Performance of the National Field Triage Guidelines Using Machine Learning: Development of a Prehospital Triage Model to Predict Severe Trauma
Source: J Med Internet Res. 2024 Sep 30;26:e58740. doi: 10.2196/58740 (PMC11474124; doi:10.2196/58740)
Supplement: Multimedia Appendix 9 [file jmir_v26i1e58740_app9.docx]

| **Characteristics** | **No critical resource use (n=182544)** | **Critical resource use (n=76604)** | ***P* value** |
| --- | --- | --- | --- |
| Sex |  |  |  |
| * Male | 106119(58.14) | 52288(68.26) | <.001 |
| * Female | 76418(41.86) | 24308(31.74) |  |
| * Total | 182537(100.00) | 76596(100.00) |  |
| Transport mode |  |  |  |
| * Ground | 172629(94.57) | 65609(85.65) | <.001 |
| * Helicopter | 9644(5.28) | 10712(13.98) |  |
| * Fixed-wing | 271(0.15) | 283(0.37) |  |
| * Total | 182544(100.00) | 76604(100.00) |  |
| Trauma center level |  |  |  |
| * Level 1 | 82347(58.30) | 34470(60.42) | <.001 |
| * Level 2 | 54837(38.82) | 20456(35.86) |  |
| * Level 3 | 4066(2.88) | 2124(3.72) |  |
| * Total | 141250(100.00) | 57050(100.00) |  |
| TCCPEN |  |  |  |
| * No | 175825(96.32) | 72700(94.90) | <.001 |
| * Yes | 6719(3.68) | 3904(5.10) |  |
| * Total | 182544(100.00) | 76604(100.00) |  |
| TCCCHEST |  |  |  |
| * No | 182097(99.76) | 75281(98.27) | <.001 |
| * Yes | 447(0.24) | 1323(1.73) |  |
| * Total | 182544(100.00) | 76604(100.00) |  |
| TCCLONGBONE |  |  |  |
| * No | 181504(99.43) | 75864(99.03) | <.001 |
| * Yes | 1040(0.57) | 740(0.97) |  |
| * Total | 182544(100.00) | 76604(100.00) |  |
| TCCCRUSHED |  |  |  |
| * No | 181713(99.54) | 76138(99.39) | <.001 |
| * Yes | 831(0.46) | 466(0.61) |  |
| * Total | 182544(100.00) | 76604(100.00) |  |
| TCCAMPUTATION |  |  |  |
| * No | 182411(99.93) | 76378(99.70) | <.001 |
| * Yes | 133(0.07) | 226(0.30) |  |
| * Total | 182544(100.00) | 76604(100.00) |  |
| TCCPELVIC |  |  |  |
| * No | 181206(99.27) | 74720(97.54) | <.001 |
| * Yes | 1338(0.73) | 1884(2.46) |  |
| * Total | 182544(100.00) | 76604(100.00) |  |
| TCCSKULLFRACTURE |  |  |  |
| * No | 182148(99.78) | 74792(97.63) | <.001 |
| * Yes | 396(0.22) | 1812(2.37) |  |
| * Total | 182544(100.00) | 76604(100.00) |  |
| TCCPARALYSIS |  |  |  |
| * No | 182067(99.74) | 75227(98.20) | <.001 |
| * Yes | 477(0.26) | 1377(1.80) |  |
| * Total | 182544(100.00) | 76604(100.00) |  |
| ISS score |  |  |  |
| * <16 | 169448(92.83) | 39723(51.85) | <.001 |
| * >=16 | 13096(7.17) | 36881(48.15) |  |
| * Total | 182544(100.00) | 76604(100.00) |  |
| RED criteria |  |  |  |
| * No | 166249(91.07) | 57044(74.47) | <.001 |
| * Yes | 16295(8.93) | 19560(25.53) |  |
| * Total | 182544(100.00) | 76604(100.00) |  |
| Age |  |  |  |
| * N(Missing) | 182544(0) | 76604(0) | <.001 |
| * Mean(SD) | 52.77(21.92) | 52.13(21.33) |  |
| * Median | 54 | 53 |  |
| * Q1,Q3 | 32.00,72.00 | 33.00,70.00 |  |
| EMSSBP |  |  |  |
| * N(Missing) | 177019(5525) | 72316(4288) | <.001 |
| * Mean(SD) | 140.88(26.76) | 136.24(32.29) |  |
| * Median | 140 | 136 |  |
| * Q1,Q3 | 124.00,157.00 | 116.00,156.00 |  |
| EMSPULSERATE |  |  |  |
| * N(Missing) | 178201(4343) | 74252(2352) | <.001 |
| * Mean(SD) | 89.91(19.10) | 93.12(23.15) |  |
| * Median | 88 | 90 |  |
| * Q1,Q3 | 77.00,100.00 | 78.00,108.00 |  |
| EMSRESPIRATORYRATE |  |  |  |
| * N(Missing) | 173338(9206) | 72246(4358) | <.001 |
| * Mean(SD) | 18.32(4.28) | 18.86(6.08) |  |
| * Median | 18 | 18 |  |
| * Q1,Q3 | 16.00,20.00 | 16.00,20.00 |  |
| EMSPULSEOXIMETRY |  |  |  |
| * N(Missing) | 149926(32618) | 62068(14536) | <.001 |
| * Mean(SD) | 96.72(4.73) | 95.10(6.98) |  |
| * Median | 98 | 97 |  |
| * Q1,Q3 | 96.00,99.00 | 94.00,98.00 |  |
| EMSGCSEYE |  |  |  |
| * N(Missing) | 174744(7800) | 72250(4354) | <.001 |
| * Mean(SD) | 3.94(0.31) | 3.43(1.07) |  |
| * Median | 4 | 4 |  |
| * Q1,Q3 | 4.00,4.00 | 3.00,4.00 |  |
| EMSGCSVERBAL |  |  |  |
| * N(Missing) | 174736(7808) | 72241(4363) | <.001 |
| * Mean(SD) | 4.81(0.55) | 4.00(1.45) |  |
| * Median | 5 | 5 |  |
| * Q1,Q3 | 5.00,5.00 | 4.00,5.00 |  |
| EMSGCSMOTOR |  |  |  |
| * N(Missing) | 174709(7835) | 72210(4394) | <.001 |
| * Mean(SD) | 5.93(0.42) | 5.17(1.62) |  |
| * Median | 6 | 6 |  |
| * Q1,Q3 | 6.00,6.00 | 5.00,6.00 |  |
| EMSTOTALGCS |  |  |  |
| * N(Missing) | 176190(6354) | 73757(2847) | <.001 |
| * Mean(SD) | 14.68(1.15) | 12.57(3.93) |  |
| * Median | 15 | 15 |  |
| * Q1,Q3 | 15.00,15.00 | 12.00,15.00 |  |
| Minutes spent in ED |  |  |  |
| * N(Missing) | 176767(5777) | 73534(3070) | <.001 |
| * Mean(SD) | 230.17(414.65) | 149.31(297.91) |  |
| * Median | 173 | 102 |  |
| * Q1,Q3 | 102.00,281.00 | 53.00,183.00 |  |
| Length of stay (days) |  |  |  |
| * N(Missing) | 180335(2209) | 75096(1508) | <.001 |
| * Mean(SD) | 4.77(5.53) | 10.36(12.99) |  |
| * Median | 4 | 7 |  |
| * Q1,Q3 | 2.00,6.00 | 4.00,12.00 |  |
| ISS score |  |  |  |
| * N(Missing) | 182544(0) | 76604(0) | <.001 |
| * Mean(SD) | 7.29(5.20) | 16.56(11.09) |  |
| * Median | 5 | 14 |  |
| * Q1,Q3 | 4.00,9.00 | 9.00,22.00 |  |
| PHI score |  |  |  |
| * N(Missing) | 164289(18255) | 66054(10550) | <.001 |
| * Mean(SD) | 0.89(1.66) | 2.63(3.03) |  |
| * Median | 0 | 3 |  |
| * Q1,Q3 | 0.00,1.00 | 0.00,4.00 |  |
| RTS score |  |  |  |
| * N(Missing) | 164814(17730) | 66325(10279) | <.001 |
| * Mean(SD) | 11.91(0.43) | 11.22(1.48) |  |
| * Median | 12 | 12 |  |
| * Q1,Q3 | 12.00,12.00 | 11.00,12.00 |  |
