# Supplementary material for: Enhancing Performance of the National Field Triage Guidelines Using Machine Learning: Development of a Prehospital Triage Model to Predict Severe Trauma
Source: J Med Internet Res. 2024 Sep 30;26:e58740. doi: 10.2196/58740 (PMC11474124; doi:10.2196/58740)
Supplement: Multimedia Appendix 10 [file jmir_v26i1e58740_app10.docx]

| **Characteristics** | **No critical resource use (n=330292)** | **Critical resource use (n=129551)** | ***P* value** |
| --- | --- | --- | --- |
| Sex |  |  |  |
| * Male | 188859(57.19) | 87990(67.93) | <.001 |
| * Female | 141399(42.81) | 41538(32.07) |  |
| * Total | 330258(100.00) | 129528(100.00) |  |
| Transport mode |  |  |  |
| * Ground | 314336(95.17) | 112291(86.68) | <.001 |
| * Helicopter | 15627(4.73) | 16926(13.07) |  |
| * Fixed-wing | 329(0.10) | 334(0.26) |  |
| * Total | 330292(100.00) | 129551(100.00) |  |
| Trauma center level |  |  |  |
| * Level 1 | 146872(57.03) | 57652(59.13) | <.001 |
| * Level 2 | 103393(40.15) | 35806(36.72) |  |
| * Level 3 | 7253(2.82) | 4048(4.15) |  |
| * Total | 257518(100.00) | 97506(100.00) |  |
| TCCPEN |  |  |  |
| * No | 319843(96.84) | 123621(95.42) | <.001 |
| * Yes | 10449(3.16) | 5930(4.58) |  |
| * Total | 330292(100.00) | 129551(100.00) |  |
| TCCCHEST |  |  |  |
| * No | 329533(99.77) | 127196(98.18) | <.001 |
| * Yes | 759(0.23) | 2355(1.82) |  |
| * Total | 330292(100.00) | 129551(100.00) |  |
| TCCLONGBONE |  |  |  |
| * No | 328542(99.47) | 128204(98.96) | <.001 |
| * Yes | 1750(0.53) | 1347(1.04) |  |
| * Total | 330292(100.00) | 129551(100.00) |  |
| TCCCRUSHED |  |  |  |
| * No | 328650(99.50) | 128589(99.26) | <.001 |
| * Yes | 1642(0.50) | 962(0.74) |  |
| * Total | 330292(100.00) | 129551(100.00) |  |
| TCCAMPUTATION |  |  |  |
| * No | 330050(99.93) | 129201(99.73) | <.001 |
| * Yes | 242(0.07) | 350(0.27) |  |
| * Total | 330292(100.00) | 129551(100.00) |  |
| TCCPELVIC |  |  |  |
| * No | 327912(99.28) | 126337(97.52) | <.001 |
| * Yes | 2380(0.72) | 3214(2.48) |  |
| * Total | 330292(100.00) | 129551(100.00) |  |
| TCCSKULLFRACTURE |  |  |  |
| * No | 329499(99.76) | 126315(97.50) | <.001 |
| * Yes | 793(0.24) | 3236(2.50) |  |
| * Total | 330292(100.00) | 129551(100.00) |  |
| TCCPARALYSIS |  |  |  |
| * No | 329505(99.76) | 127317(98.28) | <.001 |
| * Yes | 787(0.24) | 2234(1.72) |  |
| * Total | 330292(100.00) | 129551(100.00) |  |
| ISS score |  |  |  |
| * <16 | 308220(93.32) | 67035(51.74) | <.001 |
| * >=16 | 22072(6.68) | 62516(48.26) |  |
| * Total | 330292(100.00) | 129551(100.00) |  |
| RED criteria |  |  |  |
| * No | 302755(91.66) | 98019(75.66) | <.001 |
| * Yes | 27537(8.34) | 31532(24.34) |  |
| * Total | 330292(100.00) | 129551(100.00) |  |
| Age |  |  |  |
| * N(Missing) | 330292(0) | 129551(0) | <.001 |
| * Mean(SD) | 53.93(21.87) | 53.34(21.29) |  |
| * Median | 56 | 55 |  |
| * Q1,Q3 | 34.00,73.00 | 34.00,72.00 |  |
| EMSSBP |  |  |  |
| * N(Missing) | 321116(9176) | 122193(7358) | <.001 |
| * Mean(SD) | 141.75(27.06) | 136.87(32.54) |  |
| * Median | 140 | 137 |  |
| * Q1,Q3 | 124.00,158.00 | 116.00,157.00 |  |
| EMSPULSERATE |  |  |  |
| * N(Missing) | 323477(6815) | 125954(3597) | <.001 |
| * Mean(SD) | 89.74(19.31) | 92.90(23.31) |  |
| * Median | 88 | 90 |  |
| * Q1,Q3 | 76.00,100.00 | 77.00,107.00 |  |
| EMSRESPIRATORYRATE |  |  |  |
| * N(Missing) | 314440(15852) | 122563(6988) | <.001 |
| * Mean(SD) | 18.35(4.35) | 18.92(6.06) |  |
| * Median | 18 | 18 |  |
| * Q1,Q3 | 16.00,20.00 | 16.00,20.00 |  |
| EMSPULSEOXIMETRY |  |  |  |
| * N(Missing) | 278245(52047) | 107584(21967) | <.001 |
| * Mean(SD) | 96.65(4.68) | 94.92(7.12) |  |
| * Median | 98 | 97 |  |
| * Q1,Q3 | 96.00,99.00 | 94.00,98.00 |  |
| EMSGCSEYE |  |  |  |
| * N(Missing) | 317233(13059) | 123070(6481) | <.001 |
| * Mean(SD) | 3.95(0.31) | 3.45(1.06) |  |
| * Median | 4 | 4 |  |
| * Q1,Q3 | 4.00,4.00 | 4.00,4.00 |  |
| EMSGCSVERBAL |  |  |  |
| * N(Missing) | 317214(13078) | 123070(6481) | <.001 |
| * Mean(SD) | 4.81(0.56) | 4.01(1.43) |  |
| * Median | 5 | 5 |  |
| * Q1,Q3 | 5.00,5.00 | 4.00,5.00 |  |
| EMSGCSMOTOR |  |  |  |
| * N(Missing) | 317195(13097) | 123039(6512) | <.001 |
| * Mean(SD) | 5.93(0.42) | 5.19(1.60) |  |
| * Median | 6 | 6 |  |
| * Q1,Q3 | 6.00,6.00 | 5.00,6.00 |  |
| EMSTOTALGCS |  |  |  |
| * N(Missing) | 318157(12135) | 124016(5535) | <.001 |
| * Mean(SD) | 14.69(1.13) | 12.64(3.89) |  |
| * Median | 15 | 15 |  |
| * Q1,Q3 | 15.00,15.00 | 12.00,15.00 |  |
| Minutes spent in ED |  |  |  |
| * N(Missing) | 322141(8151) | 125739(3812) | <.001 |
| * Mean(SD) | 233.68(1652.57) | 142.36(352.20) |  |
| * Median | 172.2 | 99 |  |
| * Q1,Q3 | 103.20,279.00 | 52.20,175.20 |  |
| Length of stay (days) |  |  |  |
| * N(Missing) | 326525(3767) | 127647(1904) | <.001 |
| * Mean(SD) | 4.77(5.41) | 10.52(13.02) |  |
| * Median | 4 | 7 |  |
| * Q1,Q3 | 2.00,6.00 | 4.00,12.00 |  |
| ISS score |  |  |  |
| * N(Missing) | 330292(0) | 129551(0) | <.001 |
| * Mean(SD) | 7.21(5.13) | 16.60(11.03) |  |
| * Median | 5 | 14 |  |
| * Q1,Q3 | 4.00,9.00 | 9.00,22.00 |  |
| PHI score |  |  |  |
| * N(Missing) | 299315(30977) | 112593(16958) | <.001 |
| * Mean(SD) | 0.90(1.68) | 2.62(3.03) |  |
| * Median | 0 | 3 |  |
| * Q1,Q3 | 0.00,1.00 | 0.00,4.00 |  |
| RTS score |  |  |  |
| * N(Missing) | 300202(30090) | 113010(16541) | <.001 |
| * Mean(SD) | 11.90(0.43) | 11.23(1.46) |  |
| * Median | 12 | 12 |  |
| * Q1,Q3 | 12.00,12.00 | 11.00,12.00 |  |
