# Supplementary material for: Enhancing Performance of the National Field Triage Guidelines Using Machine Learning: Development of a Prehospital Triage Model to Predict Severe Trauma
Source: J Med Internet Res. 2024 Sep 30;26:e58740. doi: 10.2196/58740 (PMC11474124; doi:10.2196/58740)
Supplement: Multimedia Appendix 11 [file jmir_v26i1e58740_app11.docx]

| **Prediction Tool** | **Specificity** | **Sensitivity** | **Accuracy** | **Undertriage rate**  **(1-NPV)** | **Overtriage rate**  **(1-PPV)** | **Youden index** |
| --- | --- | --- | --- | --- | --- | --- |
| **Training set** | |  |  |  |  |  |
| pTEST | 0.500 | 0.774(0.772-0.776) | 0.580(0.580-0.581) | 0.158(0.157-0.159) | 0.609(0.608-0.609) | 1.274 |
| GCS | 0.500 | 0.678(0.677-0.679) | 0.552(0.551-0.552) | 0.209(0.208-0.209) | 0.643(0.643-0.643) | 1.178 |
| PHI | 0.500 | 0.706(0.704-0.708) | 0.559(0.558-0.559) | 0.189(0.188-0.190) | 0.640(0.640-0.641) | 1.206 |
| RTS | 0.500 | 0.633(0.632-0.634) | 0.538(0.538-0.538) | 0.226(0.225-0.227) | 0.665(0.665-0.665) | 1.133 |
| RED criteria | 0.500 | 0.593(0.592-0.594) | 0.527(0.527-0.528) | 0.253(0.252-0.253) | 0.670(0.669-0.670) | 1.093 |
| **Internal validation set** | |  |  |  |  |  |
| pTEST | 0.500 | 0.771(0.768-0.774) | 0.580(0.579-0.581) | 0.161(0.160-0.163) | 0.607(0.606-0.608) | 1.271 |
| GCS | 0.500 | 0.679(0.676-0.681) | 0.552(0.552-0.553) | 0.210(0.209-0.211) | 0.641(0.640-0.641) | 1.179 |
| PHI | 0.500 | 0.706(0.703-0.709) | 0.559(0.558-0.560) | 0.191(0.190-0.193) | 0.638(0.637-0.639) | 1.206 |
| RTS | 0.500 | 0.632(0.631-0.635) | 0.538(0.538-0.539) | 0.228(0.227-0.229) | 0.663(0.662-0.663) | 1.132 |
| RED criteria | 0.500 | 0.591(0.590-0.593) | 0.527(0.527-0.527) | 0.255(0.255-0.256) | 0.668(0.668-0.669) | 1.091 |
| **External validation set** | |  |  |  |  |  |
| pTEST | 0.500 | 0.772(0.770-0.774) | 0.577(0.576-0.577) | 0.152(0.150-0.153) | 0.623(0.622-0.624) | 1.272 |
| GCS | 0.500 | 0.677(0.676-0.679) | 0.550(0.549-0.550) | 0.200(0.199-0.201) | 0.656(0.655-0.656) | 1.177 |
| PHI | 0.500 | 0.705(0.703-0.707) | 0.556(0.555-0.557) | 0.182(0.181-0.183) | 0.653(0.653-0.654) | 1.205 |
| RTS | 0.500 | 0.631(0.630-0.633) | 0.536(0.536-0.536) | 0.217(0.217-0.218) | 0.678(0.677-0.678) | 1.131 |
| RED criteria | 0.500 | 0.587(0.586-0.589) | 0.525(0.524-0.525) | 0.245(0.244-0.245) | 0.685(0.684-0.685) | 1.087 |
