# Supplementary material for: Enhancing Performance of the National Field Triage Guidelines Using Machine Learning: Development of a Prehospital Triage Model to Predict Severe Trauma
Source: J Med Internet Res. 2024 Sep 30;26:e58740. doi: 10.2196/58740 (PMC11474124; doi:10.2196/58740)
Supplement: Multimedia Appendix 12 [file jmir_v26i1e58740_app12.docx]

Multimedia Appendix 12. Model performance metrics for predicting severe trauma at the best thresholds with maximum Youden index

| **Prediction Tool** | **Best thresholds** | **Specificity** | **Sensitivity** | **Accuracy** | **Undertriage rate**  **(1-NPV)** | **Overtriage rate**  **(1-PPV)** | **Youden index** |
| --- | --- | --- | --- | --- | --- | --- | --- |
| **Training set** | |  |  |  |  |  |  |
| pTEST | ≥0.1822 | 0.806(0.782-0.820) | 0.589(0.574-0.612) | 0.767(0.752-0.777) | 0.099(0.097-0.101) | 0.604(0.590-0.622) | 1.394 |
| GCS | ≤14 | 0.824(0.823-0.825) | 0.477(0.473-0.479) | 0.763(0.762-0.764) | 0.120(0.119-0.120) | 0.634(0.632-0.636) | 1.3 |
| PHI | ≥1 | 0.710(0.709-0.712) | 0.589(0.586-0.592) | 0.690(0.689-0.691) | 0.107(0.107-0.108) | 0.703(0.702-0.704) | 1.299 |
| RTS | ≤11 | 0.916(0.915-0.916) | 0.330(0.327-0.332) | 0.815(0.814-0.816) | 0.132(0.132-0.133) | 0.552(0.548-0.555) | 1.245 |
| RED criteria | ≥1 | 0.909(0.908-0.909) | 0.309(0.307-0.312) | 0.802(0.801-0.803) | 0.141(0.141-0.142) | 0.577(0.573-0.580) | 1.218 |
| **Internal validation set** | |  |  |  |  |  |  |
| pTEST | ≥0.1822 | 0.804(0.803-0.806) | 0.585(0.581-0.588) | 0.765(0.763-0.767) | 0.100(0.100-0.101) | 0.607(0.605-0.610) | 1.389 |
| GCS | ≤14 | 0.823(0.822-0.825) | 0.477(0.471-0.481) | 0.762(0.761-0.764) | 0.120(0.119-0.121) | 0.634(0.631-0.638) | 1.301 |
| PHI | ≥1 | 0.709(0.708-0.711) | 0.588(0.584-0.593) | 0.688(0.687-0.690) | 0.108(0.107-0.109) | 0.704(0.702-0.706) | 1.297 |
| RTS | ≤11 | 0.915(0.914-0.916) | 0.329(0.324-0.335) | 0.814(0.813-0.816) | 0.132(0.131-0.133) | 0.554(0.547-0.558) | 1.244 |
| RED criteria | ≥1 | 0.909(0.908-0.910) | 0.309(0.306-0.313) | 0.802(0.801-0.803) | 0.141(0.141-0.142) | 0.577(0.572-0.580) | 1.218 |
| **External validation set** | |  |  |  |  |  |  |
| pTEST | ≥0.1822 | 0.804(0.803-0.805) | 0.585(0.582-0.589) | 0.767(0.765-0.768) | 0.096(0.095-0.097) | 0.619(0.617-0.621) | 1.389 |
| GCS | ≤14 | 0.824(0.822-0.825) | 0.474(0.471-0.478) | 0.764(0.763-0.766) | 0.115(0.115-0.116) | 0.646(0.643-0.648) | 1.298 |
| PHI | ≥1 | 0.709(0.707-0.710) | 0.588(0.584-0.591) | 0.689(0.688-0.690) | 0.103(0.102-0.104) | 0.715(0.713-0.716) | 1.296 |
| RTS | ≤11 | 0.915(0.914-0.916) | 0.328(0.324-0.332) | 0.818(0.817-0.819) | 0.127(0.126-0.128) | 0.568(0.563-0.572) | 1.243 |
| RED criteria | ≥1 | 0.914(0.913-0.915) | 0.298(0.295-0.301) | 0.809(0.808-0.810) | 0.137(0.136-0.137) | 0.583(0.580-0.587) | 1.212 |
