# Supplementary material for: Enhancing Performance of the National Field Triage Guidelines Using Machine Learning: Development of a Prehospital Triage Model to Predict Severe Trauma
Source: J Med Internet Res. 2024 Sep 30;26:e58740. doi: 10.2196/58740 (PMC11474124; doi:10.2196/58740)
Supplement: Multimedia Appendix 13 [file jmir_v26i1e58740_app13.docx]

| **Prediction Tool** | **Best thresholds** | **Specificity** | **Sensitivity** | **Accuracy** | **Undertriage rate**  **(1-NPV)** | **Overtriage rate**  **(1-PPV)** | **Youden index** |
| --- | --- | --- | --- | --- | --- | --- | --- |
| **Training set** | |  |  |  |  |  |  |
| pTEST | ≥0.2942 | 0.805(0.789-0.812) | 0.557(0.549-0.572) | 0.732(0.725-0.735) | 0.186(0.184-0.188) | 0.458(0.452-0.470) | 1.362 |
| GCS | ≤14 | 0.847(0.846-0.848) | 0.455(0.452-0.457) | 0.733(0.732-0.734) | 0.209(0.208-0.210) | 0.451(0.449-0.454) | 1.301 |
| PHI | ≥1 | 0.731(0.730-0.732) | 0.571(0.568-0.573) | 0.686(0.684-0.686) | 0.189(0.188-0.190) | 0.542(0.541-0.544) | 1.302 |
| RTS | ≤11 | 0.936(0.935-0.936) | 0.313(0.311-0.316) | 0.759(0.758-0.760) | 0.226(0.225-0.227) | 0.340(0.337-0.343) | 1.249 |
| RED criteria | ≥1 | 0.912(0.911-0.913) | 0.259(0.257-0.261) | 0.720(0.719-0.721) | 0.253(0.252-0.253) | 0.450(0.448-0.453) | 1.171 |
| **Internal validation set** | |  |  |  |  |  |  |
| pTEST | ≥0.2942 | 0.802(0.801-0.804) | 0.552(0.548-0.555) | 0.728(0.727-0.730) | 0.190(0.189-0.191) | 0.461(0.458-0.463) | 1.354 |
| GCS | ≤14 | 0.846(0.845-0.848) | 0.456(0.452-0.460) | 0.732(0.731-0.734) | 0.210(0.209-0.211) | 0.449(0.446-0.452) | 1.302 |
| PHI | ≥1 | 0.731(0.729-0.733) | 0.570(0.567-0.575) | 0.685(0.684-0.687) | 0.191(0.190-0.192) | 0.540(0.538-0.542) | 1.301 |
| RTS | ≤11 | 0.935(0.934-0.937) | 0.312(0.308-0.316) | 0.756(0.755-0.758) | 0.228(0.227-0.229) | 0.340(0.335-0.346) | 1.248 |
| RED criteria | ≥1 | 0.911(0.910-0.912) | 0.255(0.252-0.258) | 0.717(0.716-0.718) | 0.255(0.255-0.256) | 0.454(0.450-0.459) | 1.166 |
| **External validation set** | |  |  |  |  |  |  |
| pTEST | ≥0.2942 | 0.801(0.800-0.802) | 0.556(0.553-0.558) | 0.732(0.731-0.733) | 0.178(0.178-0.179) | 0.477(0.475-0.479) | 1.357 |
| GCS | ≤14 | 0.847(0.845-0.848) | 0.453(0.451-0.455) | 0.737(0.736-0.738) | 0.200(0.200-0.201) | 0.466(0.463-0.468) | 1.300 |
| PHI | ≥1 | 0.729(0.727-0.731) | 0.570(0.566-0.572) | 0.686(0.684-0.687) | 0.182(0.181-0.183) | 0.558(0.557-0.560) | 1.299 |
| RTS | ≤11 | 0.934(0.933-0.935) | 0.312(0.310-0.314) | 0.764(0.763-0.764) | 0.217(0.217-0.218) | 0.361(0.358-0.365) | 1.245 |
| RED criteria | ≥1 | 0.917(0.916-0.918) | 0.243(0.241-0.246) | 0.727(0.726-0.728) | 0.245(0.244-0.245) | 0.466(0.462-0.470) | 1.160 |
