# Supplementary material for: Enhancing Performance of the National Field Triage Guidelines Using Machine Learning: Development of a Prehospital Triage Model to Predict Severe Trauma
Source: J Med Internet Res. 2024 Sep 30;26:e58740. doi: 10.2196/58740 (PMC11474124; doi:10.2196/58740)
Supplement: Multimedia Appendix 15 [file jmir_v26i1e58740_app15.docx]

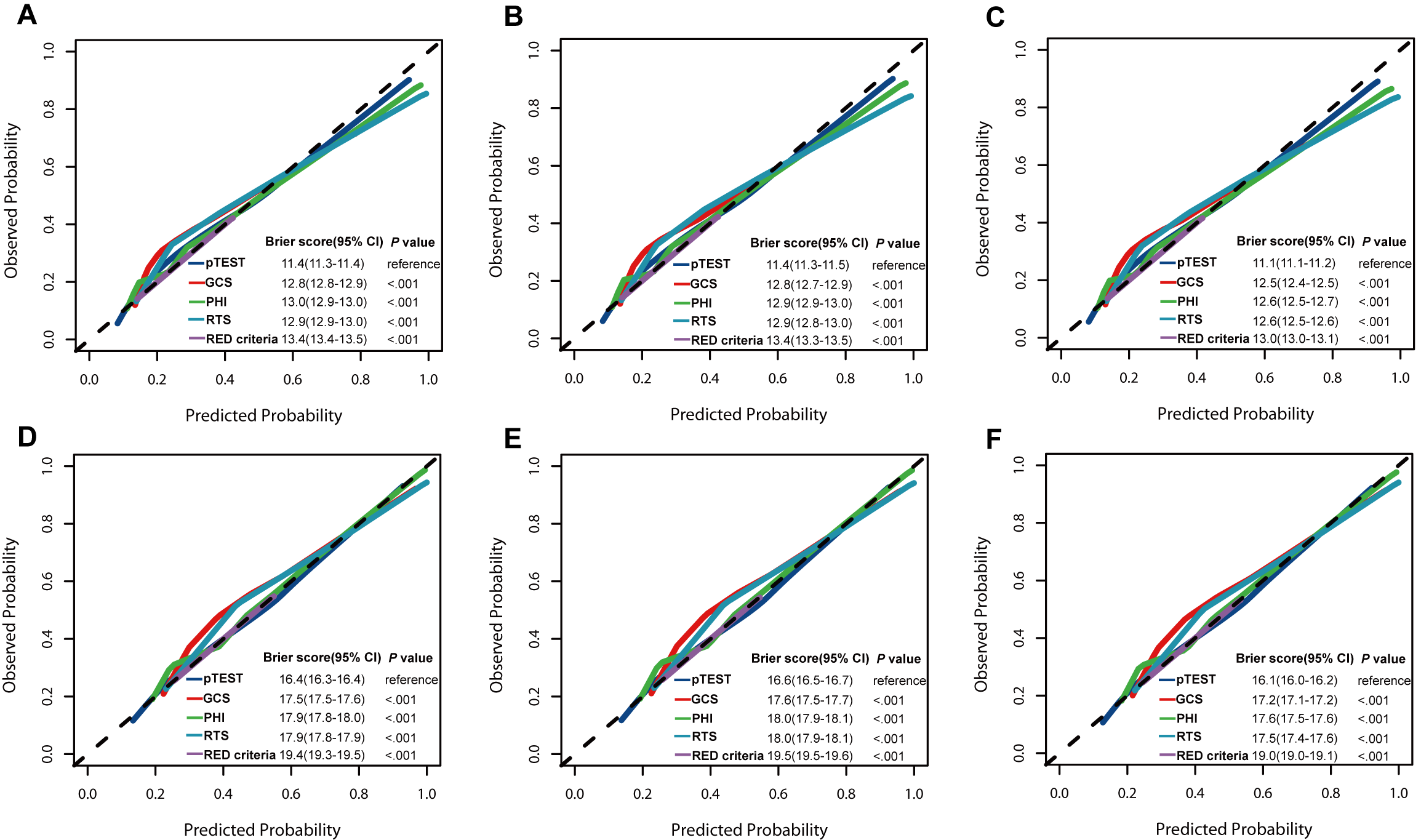


Multimedia Appendix 15. Calibration curves of five models. (A) Predicting severe trauma in training set. (B) Predicting severe trauma in internal validation set. (C) Predicting severe trauma in external validation set. (D) Predicting critical resource use in training set. (E) Predicting critical resource use in internal validation set. (F) Predicting critical resource use in external validation set.
