# Supplementary material for: Enhancing Performance of the National Field Triage Guidelines Using Machine Learning: Development of a Prehospital Triage Model to Predict Severe Trauma
Source: J Med Internet Res. 2024 Sep 30;26:e58740. doi: 10.2196/58740 (PMC11474124; doi:10.2196/58740)
Supplement: Multimedia Appendix 16 [file jmir_v26i1e58740_app16.docx]

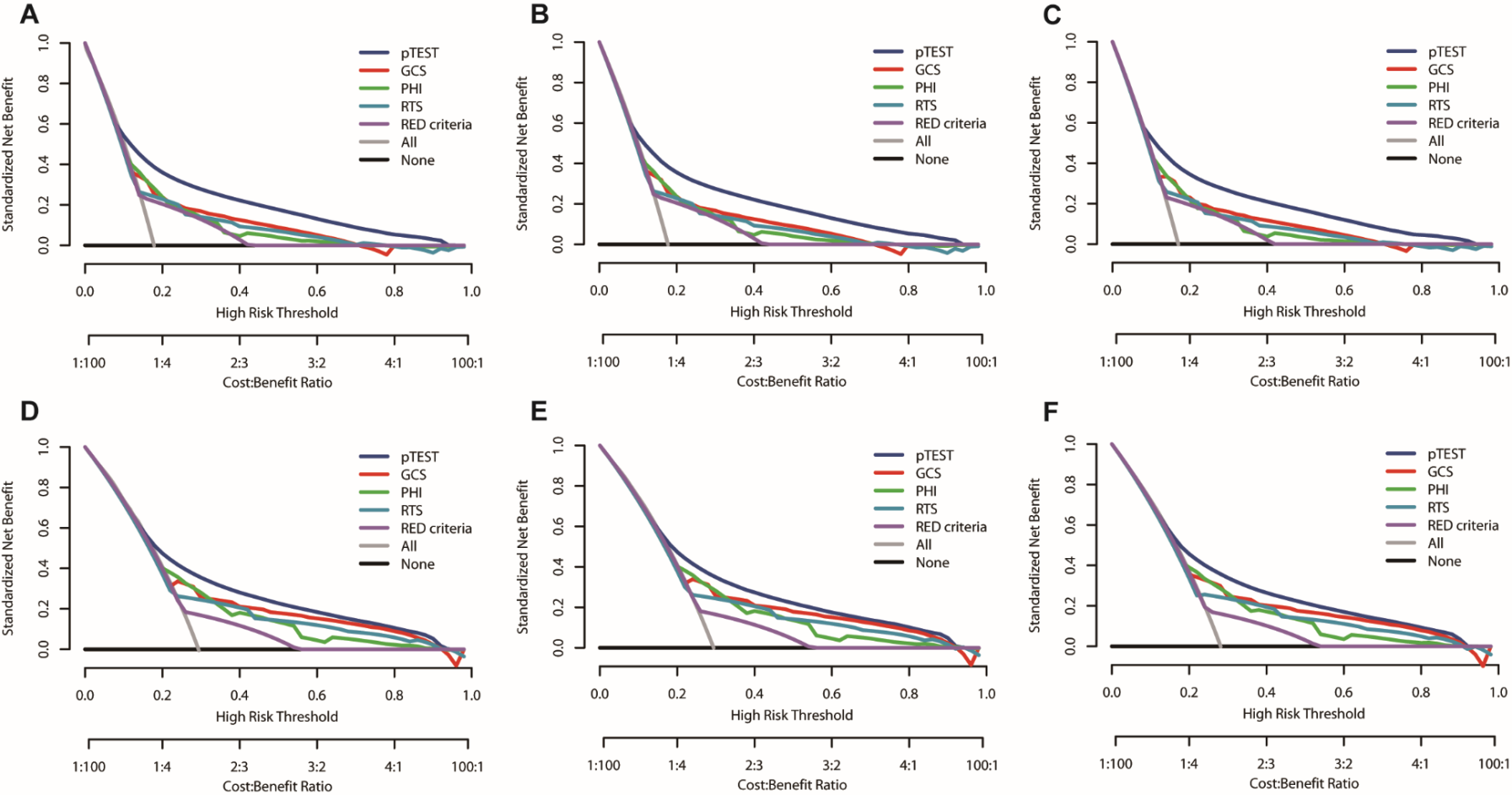


Multimedia Appendix S16. DCA curves of 5 models. (A) Predicting severe trauma in training set. (B) Predicting severe trauma in internal validation set. (C) Predicting severe trauma in external validation set. (D) Predicting critical resource use in training set. (E) Predicting critical resource use in internal validation set. (F) Predicting critical resource use in external validation set. For example, in the training set (A), for a decision threshold 0.1822 (best threshold in ROC) of severe trauma probability, the pTEST would identify 38 additional severe traumas per 100 patients (net benefit 0.38) compared with the strategy “intervention for none,” without increasing the number of false positives. In addition, compared with the strategy “intervention for all,” pTEST would avoid 42 false positives for unnecessary overtriage per 100 patients [net benefit=0.38–(–0.04) = 0.42] with an 18.3% threshold, without decreasing the number of true positives.
