# Supplementary material for: Enhancing Performance of the National Field Triage Guidelines Using Machine Learning: Development of a Prehospital Triage Model to Predict Severe Trauma
Source: J Med Internet Res. 2024 Sep 30;26:e58740. doi: 10.2196/58740 (PMC11474124; doi:10.2196/58740)
Supplement: Multimedia Appendix 17 [file jmir_v26i1e58740_app17.docx]

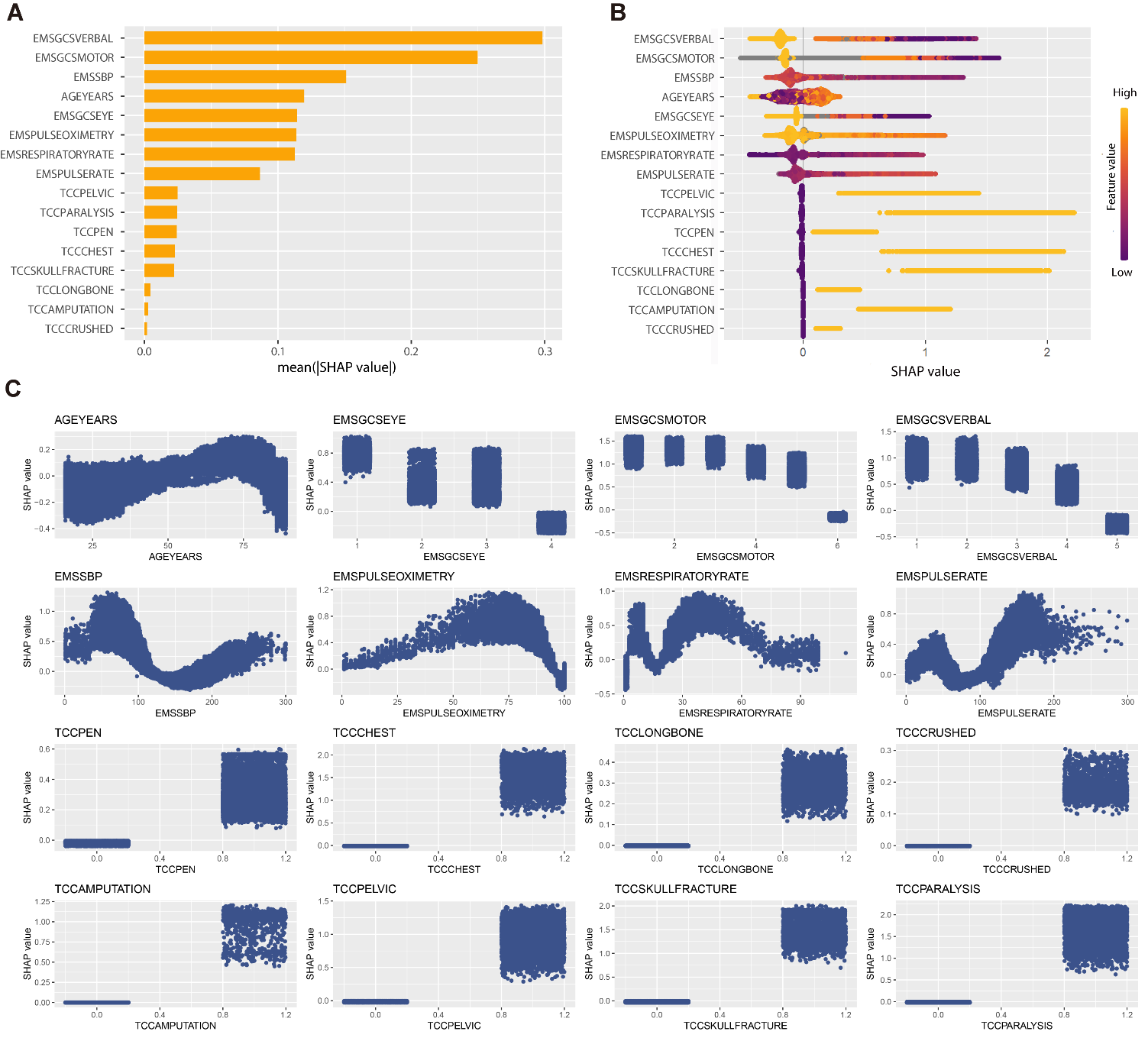


Multimedia Appendix S17. Global model explanation for predicting critical resource use by the SHAP method in training set. (A) SHAP summary bar plot of the average SHAP value for each variable. (B) SHAP summary dot plot. In each variable, a dot is made for each single patient, representing the SHAP value of this variable. The colors of the dots demonstrate the actual values of the features, and the dots are stacked vertically to show density. (C) SHAP dependence plot. Each dependence plot shows the association between the actual value and the SHAP value of the variable, and each dot represents a single patient.
