# Supplementary material for: Enhancing Performance of the National Field Triage Guidelines Using Machine Learning: Development of a Prehospital Triage Model to Predict Severe Trauma
Source: J Med Internet Res. 2024 Sep 30;26:e58740. doi: 10.2196/58740 (PMC11474124; doi:10.2196/58740)
Supplement: Multimedia Appendix 18 [file jmir_v26i1e58740_app18.docx]

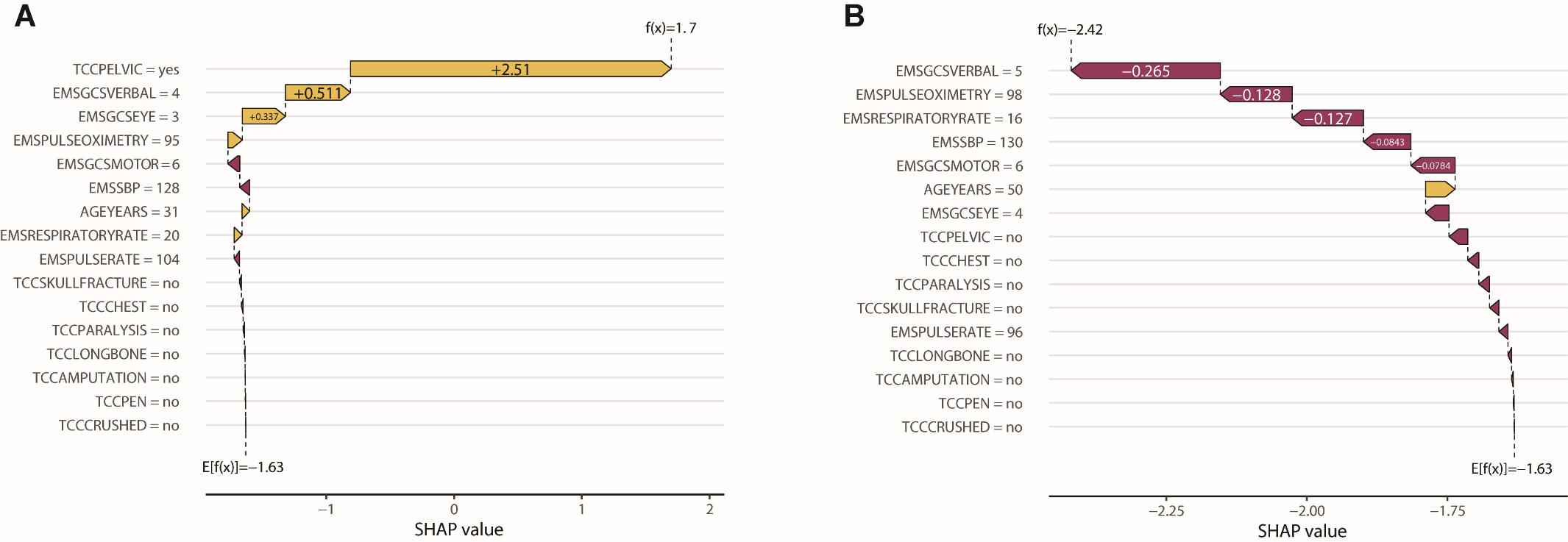


Multimedia Appendix S18. Local model explanation for predicting severe trauma by the SHAP method in training set. (A) Waterfall plot of risks contributed by each variable for a patient at high risk of severe trauma. (B) Waterfall plot for a patient at low risk of severe trauma. The prediction starts from the average of all prediction. The contribution of each variable is shown as an arrow, and the leftward and purple arrows indicate a decrease (negative SHAP value) in the probability of severe trauma, the rightward and yellow arrows indicate a increase. The length of each arrow is proportional to the SHAP value for a given feature. In the patient with severe trauma (A), TCCPELVIC (yes), EMSGCSVERBAL (4), EMSGCSEYE (3), EMSPULSEOXIMETRY (95), AGEYEARS (31), and EMSRESPIRATORYRATE (20) drove high-risk prediction against EMSGCSMOTOR (6), EMSSBP (128), and eight other variables. In the patient without severe trauma (B), EMSGCSVERBAL (5), EMSPULSEOXIMETRY (98), EMSRESPIRATORYRATE (16), EMSSBP (130), EMSGCSMOTOR (6), and 10 other variables decreased the risk of AGEYEARS (50) risk.
