# Supplementary material for: Enhancing Performance of the National Field Triage Guidelines Using Machine Learning: Development of a Prehospital Triage Model to Predict Severe Trauma
Source: J Med Internet Res. 2024 Sep 30;26:e58740. doi: 10.2196/58740 (PMC11474124; doi:10.2196/58740)
Supplement: Multimedia Appendix 19 [file jmir_v26i1e58740_app19.docx]

| **Subgroups** | **Total Patients, No.** | **Patients With severe trauma, No. (%)** | **Estimate (95%CI)** | | |
| --- | --- | --- | --- | --- | --- |
|  |  |  | **AUC** | **Undertriage rate** | **Overtriage rate** |
| **Training set** |  |  |  |  |  |
| All | 672309 | 119690(17.80) | 0.755(0.753-0.757) | 0.080(0.079-0.081) | 0.743(0.742-0.744) |
| Age |  |  |  |  |  |
| <60 years old | 385598 | 76621(19.87) | 0.773(0.771-0.775) | 0.081(0.080-0.082) | 0.710(0.710-0.711) |
| ≥60 years old | 286711 | 43069(15.02) | 0.717(0.714-0.720) | 0.080(0.079-0.082) | 0.790(0.789-0.791) |
| Sex |  |  |  |  |  |
| Male | 403434 | 83808(20.77) | 0.754(0.752-0.756) | 0.096(0.094-0.097) | 0.705(0.704-0.706) |
| Female | 268788 | 35865(13.34) | 0.744(0.741-0.748) | 0.063(0.062-0.064) | 0.806(0.805-0.807) |
| TRANSPORT MODE |  |  |  |  |  |
| Ground | 622489 | 99264(15.95) | 0.744(0.742-0.746) | 0.075(0.074-0.076) | 0.770(0.770-0.771) |
| Helicopter | 48464 | 19891(41.04) | 0.750(0.745-0.754) | 0.216(0.210-0.221) | 0.472(0.470-0.474) |
| Trauma type |  |  |  |  |  |
| Blunt | 594679 | 105084(17.67) | 0.749(0.747-0.750) | 0.082(0.081-0.083) | 0.746(0.746-0.747) |
| Penetrating | 61171 | 11654(19.05) | 0.815(0.810-0.820) | 0.059(0.056-0.062) | 0.710(0.708-0.712) |
| Prehospital time |  |  |  |  |  |
| <60 minutes | 523870 | 91536(17.47) | 0.761(0.759-0.763) | 0.075(0.074-0.076) | 0.745(0.744-0.746) |
| ≥60 minutes | 97185 | 18256(18.78) | 0.733(0.728-0.737) | 0.098(0.095-0.100) | 0.738(0.737-0.740) |
| **Internal validation set** |  |  |  |  |  |
| All | 288134 | 51296(17.80) | 0.751(0.749-0.754) | 0.082(0.081-0.083) | 0.744(0.743-0.745) |
| Age |  |  |  |  |  |
| <60 years old | 164754 | 32852(19.94) | 0.770(0.767-0.773) | 0.083(0.082-0.085) | 0.711(0.710-0.712) |
| ≥60 years old | 123380 | 18444(14.95) | 0.712(0.708-0.717) | 0.081(0.079-0.084) | 0.792(0.790-0.794) |
| Sex |  |  |  |  |  |
| Male | 172821 | 35933(20.79) | 0.751(0.748-0.754) | 0.098(0.096-0.100) | 0.706(0.705-0.707) |
| Female | 115297 | 15358(13.32) | 0.741(0.736-0.745) | 0.064(0.062-0.065) | 0.807(0.806-0.808) |
| TRANSPORT MODE |  |  |  |  |  |
| Ground | 266622 | 42479(15.93) | 0.739(0.736-0.741) | 0.077(0.076-0.078) | 0.772(0.771-0.773) |
| Helicopter | 20911 | 8584(41.05) | 0.756(0.749-0.762) | 0.210(0.203-0.219) | 0.470(0.468-0.473) |
| Trauma type |  |  |  |  |  |
| Blunt | 255053 | 45153(17.70) | 0.746(0.743-0.749) | 0.084(0.082-0.085) | 0.747(0.746-0.748) |
| Penetrating | 25989 | 4848(18.65) | 0.810(0.802-0.817) | 0.060(0.056-0.065) | 0.717(0.715-0.720) |
| Prehospital time |  |  |  |  |  |
| <60 minutes | 225004 | 39367(17.50) | 0.757(0.754-0.760) | 0.078(0.077-0.079) | 0.746(0.746-0.747) |
| ≥60 minutes | 41523 | 7775(18.72) | 0.730(0.723-0.736) | 0.098(0.095-0.101) | 0.740(0.737-0.741) |
| **External validation set** |  |  |  |  |  |
| All | 508703 | 86902(17.08) | 0.750(0.748-0.752) | 0.078(0.077-0.079) | 0.753(0.753-0.754) |
| Age |  |  |  |  |  |
| <60 years old | 276495 | 53693(19.42) | 0.771(0.769-0.774) | 0.079(0.078-0.081) | 0.716(0.716-0.717) |
| ≥60 years old | 232208 | 33209(14.30) | 0.710(0.707-0.714) | 0.078(0.077-0.080) | 0.801(0.800-0.802) |
| Sex |  |  |  |  |  |
| Male | 300711 | 60497(20.12) | 0.750(0.748-0.753) | 0.093(0.092-0.095) | 0.714(0.713-0.715) |
| Female | 207921 | 26388(12.69) | 0.738(0.735-0.742) | 0.061(0.059-0.062) | 0.816(0.815-0.817) |
| TRANSPORT MODE |  |  |  |  |  |
| Ground | 474645 | 72940(15.37) | 0.739(0.736-0.741) | 0.074(0.073-0.075) | 0.779(0.778-0.780) |
| Helicopter | 33346 | 13695(41.07) | 0.749(0.743-0.754) | 0.218(0.211-0.226) | 0.473(0.470-0.476) |
| Trauma type |  |  |  |  |  |
| Blunt | 454555 | 77228(16.99) | 0.745(0.742-0.747) | 0.080(0.079-0.081) | 0.756(0.756-0.757) |
| Penetrating | 44280 | 8082(18.25) | 0.810(0.805-0.816) | 0.056(0.053-0.059) | 0.721(0.719-0.723) |
| Prehospital time |  |  |  |  |  |
| <60 minutes | 195958 | 32113(16.39) | 0.759(0.756-0.763) | 0.071(0.070-0.072) | 0.760(0.759-0.761) |
| ≥60 minutes | 37037 | 6890(18.60) | 0.735(0.728-0.743) | 0.094(0.090-0.097) | 0.739(0.736-0.741) |

Undertriage and overtriage rate were calculated at a fixed specificity of 0.5; Prehospital time was the time from EMS arrival at scene to ED/hospital arrival.
