# Supplementary material for: Enhancing Performance of the National Field Triage Guidelines Using Machine Learning: Development of a Prehospital Triage Model to Predict Severe Trauma
Source: J Med Internet Res. 2024 Sep 30;26:e58740. doi: 10.2196/58740 (PMC11474124; doi:10.2196/58740)
Supplement: Multimedia Appendix 20 [file jmir_v26i1e58740_app20.docx]

Multimedia Appendix 20. The performance of the pTEST model for predicting critical resource use in different subgroups

| **Subgroups** | **Total Patients, No.** | **Patients With critical resource use, No. (%)** | **Estimate (95%CI)** | | |
| --- | --- | --- | --- | --- | --- |
|  |  |  | **AUC** | **Undertriage rate** | **Overtriage rate** |
| **Training set** |  |  |  |  |  |
| All | 604806 | 177570(29.36) | 0.722(0.721-0.724) | 0.167(0.166-0.168) | 0.613(0.612-0.614) |
| Age |  |  |  |  |  |
| <60 years old | 355188 | 106036(29.85) | 0.752(0.750-0.754) | 0.147(0.146-0.149) | 0.596(0.595-0.597) |
| ≥60 years old | 249618 | 71534(28.66) | 0.682(0.680-0.685) | 0.185(0.183-0.187) | 0.635(0.633-0.636) |
| Sex |  |  |  |  |  |
| Male | 369873 | 121665(32.89) | 0.732(0.730-0.733) | 0.184(0.182-0.185) | 0.570(0.569-0.570) |
| Female | 234852 | 55878(23.79) | 0.695(0.693-0.698) | 0.146(0.145-0.148) | 0.688(0.687-0.689) |
| TRANSPORT MODE |  |  |  |  |  |
| Ground | 556333 | 152276(27.37) | 0.710(0.709-0.712) | 0.161(0.160-0.163) | 0.640(0.640-0.641) |
| Helicopter | 47203 | 24683(52.29) | 0.751(0.747-0.756) | 0.308(0.302-0.313) | 0.364(0.362-0.365) |
| Trauma type |  |  |  |  |  |
| Blunt | 532199 | 152460(28.65) | 0.715(0.714-0.717) | 0.167(0.166-0.168) | 0.624(0.623-0.625) |
| Penetrating | 57503 | 18540(32.24) | 0.780(0.776-0.785) | 0.142(0.138-0.146) | 0.560(0.558-0.562) |
| Prehospital time |  |  |  |  |  |
| <60 minutes | 471245 | 137638(29.21) | 0.729(0.727-0.731) | 0.161(0.160-0.162) | 0.612(0.612-0.613) |
| ≥60 minutes | 87069 | 25229(28.98) | 0.701(0.697-0.705) | 0.181(0.177-0.184) | 0.627(0.625-0.629) |
| **Internal validation set** |  |  |  |  |  |
| All | 259148 | 76604(29.56) | 0.720(0.718-0.722) | 0.170(0.168-0.171) | 0.612(0.611-0.612) |
| Age |  |  |  |  |  |
| <60 years old | 151793 | 45668(30.09) | 0.752(0.749-0.755) | 0.148(0.146-0.151) | 0.593(0.592-0.594) |
| ≥60 years old | 107355 | 30936(28.82) | 0.676(0.672-0.679) | 0.192(0.190-0.195) | 0.636(0.635-0.638) |
| Sex |  |  |  |  |  |
| Male | 158407 | 52288(33.01) | 0.729(0.727-0.732) | 0.185(0.183-0.188) | 0.569(0.568-0.570) |
| Female | 100726 | 24308(24.13) | 0.692(0.688-0.696) | 0.150(0.148-0.153) | 0.685(0.684-0.687) |
| TRANSPORT MODE |  |  |  |  |  |
| Ground | 238238 | 65609(27.54) | 0.707(0.705-0.710) | 0.164(0.162-0.165) | 0.639(0.638-0.640) |
| Helicopter | 20356 | 10712(52.62) | 0.754(0.748-0.761) | 0.305(0.297-0.315) | 0.359(0.357-0.362) |
| Trauma type |  |  |  |  |  |
| Blunt | 228149 | 65926(28.90) | 0.713(0.711-0.716) | 0.169(0.167-0.172) | 0.622(0.620-0.623) |
| Penetrating | 24468 | 7834(32.02) | 0.778(0.771-0.785) | 0.142(0.136-0.147) | 0.563(0.560-0.565) |
| Prehospital time |  |  |  |  |  |
| <60 minutes | 202310 | 59512(29.42) | 0.726(0.723-0.729) | 0.164(0.162-0.165) | 0.611(0.610-0.612) |
| ≥60 minutes | 37252 | 10860(29.15) | 0.699(0.693-0.705) | 0.183(0.177-0.188) | 0.625(0.622-0.628) |
| **External validation set** |  |  |  |  |  |
| All | 459843 | 129551(28.17) | 0.722(0.720-0.723) | 0.159(0.158-0.161) | 0.627(0.626-0.628) |
| Age |  |  |  |  |  |
| <60 years old | 256314 | 73514(28.68) | 0.758(0.755-0.760) | 0.136(0.135-0.139) | 0.607(0.607-0.608) |
| ≥60 years old | 203529 | 56037(27.53) | 0.678(0.675-0.680) | 0.180(0.178-0.182) | 0.649(0.648-0.650) |
| Sex |  |  |  |  |  |
| Male | 276849 | 87990(31.78) | 0.731(0.728-0.733) | 0.176(0.174-0.178) | 0.582(0.581-0.583) |
| Female | 182937 | 41538(22.71) | 0.695(0.691-0.698) | 0.138(0.136-0.140) | 0.701(0.699-0.702) |
| TRANSPORT MODE |  |  |  |  |  |
| Ground | 426627 | 112291(26.32) | 0.709(0.707-0.711) | 0.155(0.153-0.156) | 0.653(0.652-0.654) |
| Helicopter | 32553 | 16926(52.00) | 0.750(0.745-0.756) | 0.303(0.297-0.312) | 0.366(0.364-0.369) |
| Trauma type |  |  |  |  |  |
| Blunt | 408939 | 112585(27.53) | 0.714(0.712-0.716) | 0.160(0.159-0.162) | 0.637(0.637-0.638) |
| Penetrating | 41852 | 12949(30.94) | 0.789(0.784-0.794) | 0.125(0.121-0.130) | 0.571(0.569-0.573) |
| Prehospital time |  |  |  |  |  |
| <60 minutes | 171098 | 48798(28.52) | 0.722(0.720-0.725) | 0.160(0.158-0.163) | 0.622(0.621-0.623) |
| ≥60 minutes | 32585 | 9445(28.99) | 0.700(0.694-0.707) | 0.180(0.175-0.186) | 0.626(0.623-0.630) |

Undertriage and overtriage rate were calculated at a fixed specificity of 0.5; Prehospital time was the time from EMS arrival at scene to ED/hospital arrival.

Figure S2. The undertriage and overtriage rates of the pTEST in external validation set with the fixed sensitivity, specificity and sample size

|  |  | Actual ISS ≥ 16 | |  |
| --- | --- | --- | --- | --- |
|  |  | Yes | No |  |
| Predicted ISS≥ 16 | Yes | 12117 | 246721 | 258838 |
|  | No | 3144 | 246721 | 249865 |
|  |  | 15261 | 493442 | 508703 |

Sample size=508703

Sensitivity=12117/15261=0.794

Specificity=246721/493442=0.5

Undertriage rate=3144/249865=0.0126

Overtriage rate=246721/258838=0.9532
